# Quaternary stabilization of a GH2 *β*‐galactosidase from the psychrophile *A. ikkensis*, a flexible and unstable dimeric enzyme

**DOI:** 10.1002/pro.70141

**Published:** 2025-04-25

**Authors:** Jan S. Nowak, Nikoline Kruuse, Helena Ø. Rasmussen, Pengfei Tian, Julie Astono, Søren Schultz‐Nielsen, Mariane S. Thøgersen, Peter Stougaard, Jan Skov Pedersen, Daniel E. Otzen

**Affiliations:** ^1^ Interdisciplinary Nanoscience Center (iNANO) Aarhus University Aarhus Denmark; ^2^ Flagship Labs 97, Inc Cambridge Massachusetts USA; ^3^ Department of Environmental Science Aarhus University Roskilde Denmark; ^4^ Department of Chemistry Aarhus University Aarhus Denmark; ^5^ Department of Molecular Biology and Genetics Aarhus University Aarhus Denmark; ^6^ Present address: Zealand Academy of Technologies and Business Roskilde Denmark

**Keywords:** cold active enzyme, GH2 *β*‐galactosidase, low stability, oligomerization, psychrophile, quaternary structure, stabilizing mutations

## Abstract

Studies of cold‐active enzymes may elucidate the basis for low‐temperature activity and contribute to their wider application in energy‐efficient processes. Here we investigate the cold‐active GH2 *β*‐galactosidase from the psychrophilic bacterium *Alkalilactibacillus ikkensis* (AiLac). AiLac has a specific activity twice as high as its closest structural homolog (the mesophilic *Escherichia coli* GH2 *β*‐galactosidase) toward the lactose analog ONPG at room temperature and neutral pH, and shows biphasic behavior in Michaelis–Menten plots. AiLac is activated by Mg^2+^ and Na^+^ and is most effective at pH 7.0 and 30°C. However, early unfolding events are observed already at room temperature. Stability studies using intrinsic fluorescence, circular dichroism, and small‐angle x‐ray scattering (SAXS), combined with activity assays, showed AiLac to be highly sensitive to heat and urea and to be stabilized, but also inhibited, by loss of structural flexibility induced by the osmolyte trehalose. AlphaFold structure prediction combined with SAXS and flow‐induced dispersion analysis support a reversible monomer‐dimer model, suggesting structural adaptation to cold temperatures on a quaternary level. The low amount of dimeric buried surface area, high flexibility, and remarkably low chemical and thermal stability present an extreme example of cold adaptation promoted by high levels of solvent interactions. To investigate the relationship between evolution and oligomerization, we trained a generative deep learning model to successfully engineer functional variants that form stabilized dimers and tetramers by introducing high evolutionary fitness mutations at the interface, demonstrating an efficient way to explore the local sequence fitness landscape to modulate the equilibrium of oligomerization.

## 1 INTRODUCTION

Enzymes from psychrophilic organisms, for example, bacteria growing in polar regions or the depths of the oceans, often exhibit high catalytic efficiency at low temperatures as their hosts adapt to the cold at the molecular level (Feller & Gerday, 2003). Cold‐active enzymes present great potential for biocatalysis within industrial food production and preservation, detergents, textiles, and pharmaceuticals (Singh et al., 2016), where high activity at low operating temperatures may conveniently be combined with inactivation at moderately elevated temperatures. Understanding molecular and structural adaptations of psychrophilic enzymes opens a window for both discovery and bioengineering of novel enzymes with useful properties (Nowak & Otzen, 2024).

Efficient catalysis at low temperatures is attributed to a change in the way in which the rate constant (*k*_cat_) depends on temperature according to the general relationship $k_{\mathrm{cat}}\propto k_{B}T/h\times e^{-∆H^{ǂ}/\mathrm{RT}}\times e^{-∆S^{ǂ}/R}$, where Δ*H* ^‡^ and Δ*S* ^‡^ are the activation enthalpy and entropy of catalysis, respectively, *k*_B_ is Boltzmann's constant, *T* is the absolute temperature, *R* is the gas constant, and *h* is the Planck constant (Eyring, 1935). Numerous psychrophilic enzymes show lowered Δ*H* ^‡^ and increased Δ*S* ^‡^ compared to their mesophilic counterparts (Åqvist, 2017). This phenomenon is a key adaptation that decreases the otherwise exponential reduction in *k*_cat_ at lower temperatures. Structurally, this is seen as comparatively higher flexibility. While this makes the psychrophilic enzymes relatively less temperature dependent, it also makes them less stable than their meso‐ and thermophilic homologs (Fields, 2001). Indeed, several psychrophilic enzymes have been reported to lose activity at temperatures significantly lower than their global unfolding transition, emphasizing their lability toward heat and their high general dynamics (Cipolla et al., 2011; Collins et al., 2003; Feller et al., 1992).

Enhanced flexibility has been observed both locally and globally in protein structure (Feller & Gerday, 2003). Enzymes can achieve increased flexibility through various adaptations which vary from protein to protein, for example, fewer disulfide bridges, more Gly residues, fewer salt bridges on the protein surface, fewer aromatic residues in the protein core but more on the surface, and lower Pro and Arg content (D'Amico et al., 2003; Siddiqui & Cavicchioli, 2006). Furthermore, changes in subunit interface interactions or complete rearrangements of the subunits in a protein may influence stability and flexibility through intermolecular interactions and exposure to solvent (Russell et al., 1998). Indeed, there is a tendency for psychrophiles to reduce the level of subunit association compared to their meso‐ and thermophilic homologues. Thus, GH2 *β*‐Galactosidase forms an octamer in the thermophilic *Thermotoga maritima* (Míguez Amil et al., 2020), a tetramer in most mesophiles, and a dimer in the psychrophile *Arthrobacter* sp. 32cB (Rutkiewicz et al., 2019). Similar observations have been made for the ultrathermophilic triosephosphate isomerase from *Pyrococcus woesei* (a tetramer) (Walden et al., 2001) and psychrophilic Trigger Factor chaperone protein from *Psychrobacter frigidicolahas* (a monomer) (Robin et al., 2009). In both cases, their mesophilic counterparts are dimers and their unique oligomerization arrangements have been ascribed to adaptation to heat or cold, respectively. However, multimerization has also been observed in psychrophilic *β*‐glucosidase from *Exiguobacterium antarcticum* (a hexamer) and *β*‐galactosidase from *Arthrobacter* sp. C2‐2 (Skálová et al., 2005; Zanphorlin et al., 2016) (a tetramer), suggesting that a decrease in oligomerization is not a simple and universally applicable adaptation mechanism for activity at low temperatures. Furthermore, a thorough computational study of a cold‐adapted short chain dehydrogenase has shown that enzyme multimerization does not affect its temperature dependence of activation (Koenekoop et al., 2022). Therefore, it remains to be determined to what extent enzymes adapt to cold through changes in quaternary structure.

*β*‐galactosidases (lactases) are a group of enzymes capable of hydrolyzing *β*‐D‐galactosides such as lactose. Cold‐active *β*‐galactosidases are of special interest for dairy industries by allowing potentially cost‐effective production of lactose‐free milk under refrigerated conditions. In 2010, a GH2 *β*‐galactosidase (1041 aa, 119.13 kDa, here termed AiLac) from the bacterium *Alkalilactibacillus ikkensis* growing in ikaite columns in the Ikka Fjord of south‐western Greenland was discovered and reported to have a low activity optimum of 20–30°C, retaining 60% of its maximal activity at 5°C (Schmidt & Stougaard, 2010). This enzyme was predicted to be structurally homologous to GH2 *β*‐galactosidase from *Escherichia coli* (EcLac, a tetramer), which provides a well‐characterized mesophilic reference point.

The purpose of the present study was to investigate the structure and stability of AiLac to understand the molecular basis for its adaptations to high efficiency at low temperatures compared to EcLac. Specifically, we investigated how structure and activity are affected by temperature and urea. Temperature effects are crucial for understanding the stability of psychrophilic enzymes, while urea is a classic chemical denaturant extensively used to determine protein stability (Pace, 1986). Since structural flexibility is a key parameter for cold activity, we also introduced the sugar osmolyte trehalose, which inhibits the catalytic activity of enzymes through a decrease in structural flexibility (Fedorov et al., 2011; Jain & Roy, 2009; Sampedro & Uribe, 2004). We followed activity using the colorimetric substrate analog ONPG and measured both stability and structure through biophysical techniques including circular dichroism (CD), intrinsic fluorescence, SAXS, flow‐induced dispersion analysis (FIDA), and size‐exclusion chromatography with multi‐angle light scattering (SEC‐MALS). We observed that AiLac activity is sensitive to Mg^2+^ and NaCl and is only stable and active around neutral pH. AiLac is easily perturbed with chemical denaturants compared to other psychrophilic enzymes (Siddiqui Khawar et al., 2005) and forms a dimer that dissociates reversibly and without loss of activity, unlike the tetrameric structure of EcLac, which loses activity upon subunit dissociation (Juers et al., 2012). Finally, we explored the relationship between evolution and oligomerization. We hypothesized that most GH2 *β*‐galactosidases originate from mesophilic organisms and exhibit greater stability than AiLac, primarily due to their adoption of higher‐order oligomeric conformations such as tetramers. Therefore, mutations conferring high evolutionary fitness are likely to promote structural shifts in AiLac toward more stable dimers and tetramers. To investigate this, we trained a machine learning model to discern the pattern within the natural sequences of the GH2 *β*‐galactosidase family, extracted from the Uniprot100 protein database (Suzek et al., 2007). By incorporating highly evolutionary fit mutations at the structural interface, as predicted by our model, our experimental results demonstrated a significant increase in both the propensity for dimer and tetramer formation and the overall stability of AiLac. This highlights the efficiency of the evolutionary model as an efficient protein engineering method to modulate quaternary organization, which is directly related to the enzyme's temperature profile.

## 2 RESULTS

### 2.1 Activity of AiLac depends on Mg^2+^ and NaCl concentration

**FIGURE 1:**
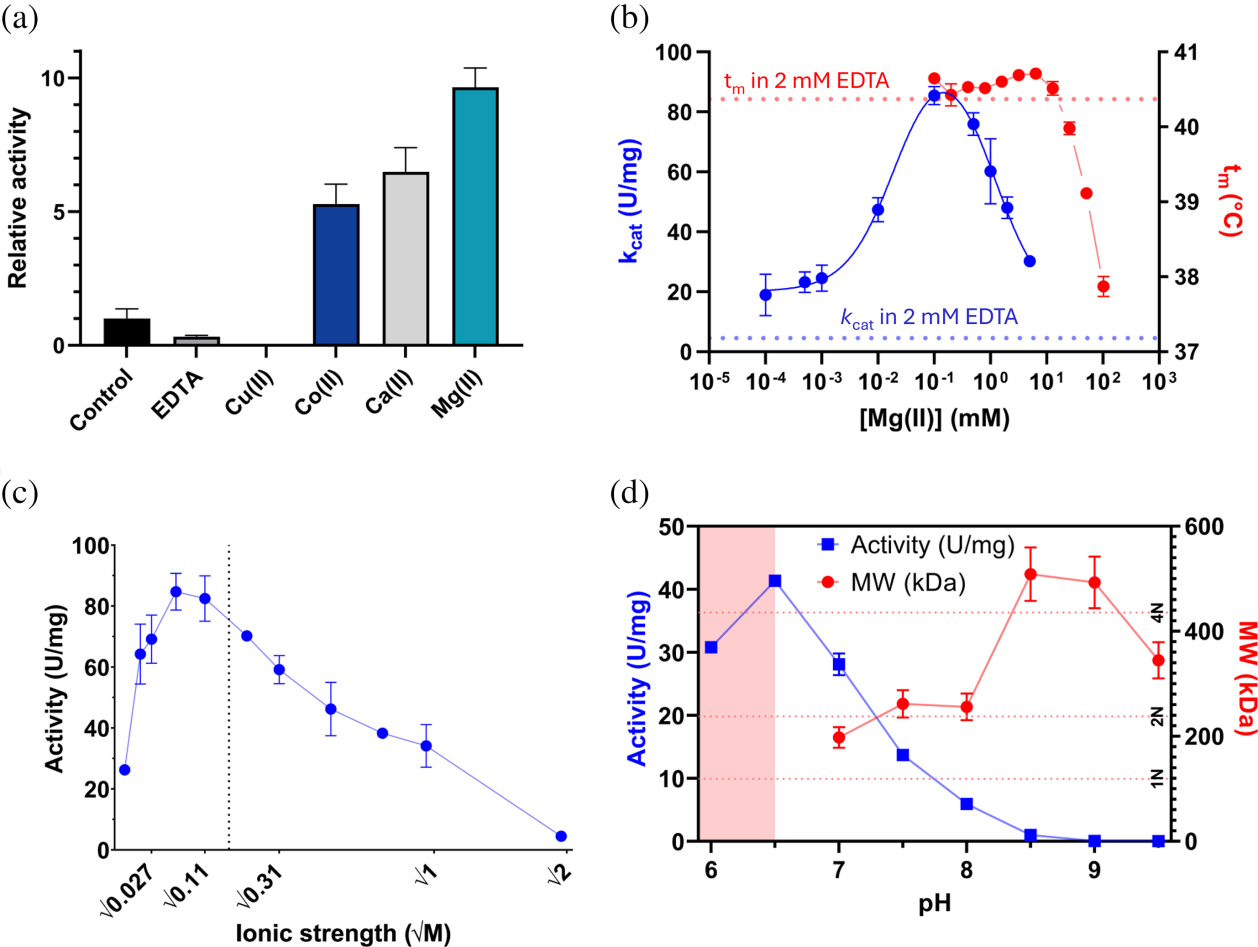
Optimal conditions for AiLac activity. (a) Effect of divalent ions and EDTA at 2 mM concentration on initial reaction rate of 8 nM AiLac in PBS. The results are normalized to the control (AiLac purified on SEC in PBS without additives). (b) Effect of Mg^2+^ on catalytic rate of 8 nM AiLac in PBS (left *y*‐axis), where the stippled blue line shows *k*_cat_ in 2 mM EDTA. Stability of 1.7 μM AiLac measured with DSF is represented by melting temperature (*t*_m_) (right *y*‐axis) where the red dotted line shows *t*_*m*_ in 2 mM EDTA. (c) Effect of NaCl on activity of 8 nM AiLac in phosphate buffer pH 7.0 with 0.1 mM Mg^2+^. Dotted line shows the ionic strength of buffer A, which is used in all subsequent experiments. d: Initial velocity (left axis, blue) and molecular weight of AiLac measured by SAXS (right axis, red) in 50 mM bis‐tris propane with pH‐values 6.0–9.5. Activity assays were performed at 8 nM concentration and SAXS at 3.4 μM. Red dotted lines mark theoretical MW of monomer (1N), dimer (2N), and tetramer (4N). AiLac aggregated at pH 6.0 and 6.5 before the SAXS experiment, represented by the red region in the graph.

To establish the basis for subsequent biophysical analyses, we first identified conditions of optimal activity for AiLac. This was done using photometric activity assays with ONPG and thermal unfolding experiments measured by far‐UV CD. GH2 *β*‐galactosidases are known to depend on Mg^2+^ for both activity and stability (Case et al., 1973; Juers et al., 2012). Therefore, we investigated metal ion effects on AiLac. Several divalent metal ions increased the activity of AiLac, including Co^2+^, Ca^2+^, and Mg^2+^; conversely, Cu^2+^ and the divalent metal ion chelator EDTA inhibited it (Figure 1a). Closer inspection using dose–response curves identified an optimum for Mg^2+^ at 0.1 mM (Figure 1b). At this optimum, *k*_cat_ is ca. 20‐fold higher than in 2 mM EDTA, namely 85.5 ± 3.0 U/mg versus 4.6 ± 0.1 U/mg (the latter shown as a blue stippled line in Figure 1b). Here, the unit of specific activity (U/mg) is defined as a μmol of product released per minute per milligram of enzyme. However, 0.1 mM Mg^2+^ does not significantly stabilize the structure in terms of melting temperature compared to 2 mM EDTA, which is indicated by the stippled red line (Figure 1b). Interestingly, titration of Mg^2+^ above 6.4 mM caused a lowering of *t*_m_. To probe the link between aggregation and increasing Mg^2+^ concentrations, we measured aggregation through dynamic light scattering (DLS) as a function of temperature in parallel with differential scanning fluorimetry (DSF). These plots unambiguously showed that AiLac aggregates at lower temperatures at higher [Mg^2+^] (Figure S1). Increased aggregation may drive irreversible unfolding, thus decreasing *t*_m_. Ionic strength (NaCl concentration) also affected activity with an optimum between 56 mM and 106 mM (Figure 1c), followed by a linear decrease with the square root of ionic strength. The linear decline in this plot suggests a simple screening effect rather than specific ion binding at the active site. To keep consistency among experiments, we decided to utilize phosphate saline (PBS) + 0.1 mM MgCl_2_ pH 7.0 (see section below for pH optimization) that has an ionic strength of 160 mM (shown with stippled black line in Figure 1c). Within error, the activity of AiLac in this buffer is the same as that measured at the peak of activity at 106 mM ionic strength (data not shown).

### 2.2 pH 7.0 provides a compromise between activity and stability

To identify the optimal pH for enzymatic activity, we carried out ONPG activity assays using the buffer Bis‐Tris Propane whose buffering capacity spans the pH range 6.0–9.5. AiLac shows the highest initial reaction rate at pH 6.5, followed by a steep decline at higher pH and no significant activity above pH 8.5–9.0 (Figure 1d). However, the enzyme aggregates at pH 6.0 and 6.5, as observed by large visible aggregates in the bottom of the Eppendorf tube occurring after ~1 h at room temperature. This shows that AiLac is only stable at or above neutral pH at RT. To probe quaternary structure, we recorded SAXS curves for 0.40 mg/mL AiLac at pH 7.0–9.5 at intervals of 0.5 pH units (Figure S2a). These data allowed us to determine the molecular weight (MW) of the AiLac structure in a model‐independent manner by extrapolating intensity to zero scattering vector modulus (*q* = 0, *I*(0)) (see “Materials and Methods”). This showed that the mass of the structure increased from mainly a dimer at pH 7.0–8.0 to a larger complex at pH 8.5 and above (Figure 1d). However, AiLac does not unfold above pH 8.5 despite complex formation; SAXS data reveal that the tertiary structure only shows minor changes, as seen from the intensity in the mid‐*q* range (Figure S2a) which is known to be largely unaffected by aggregation and oligomerization (Larsen et al., 2020). We conclude that pH 7.0 provided a compromise between high enzymatic activity and stable structure and therefore settled on phosphate buffer saline with an ionic strength of 160 mM at pH 7.0 in combination with 0.1 mM MgCl_2_ as our optimal buffer for subsequent studies.

### 2.3 AiLac has higher specific activity than EcLac at low temperatures and biphasic kinetics

**FIGURE 2:**
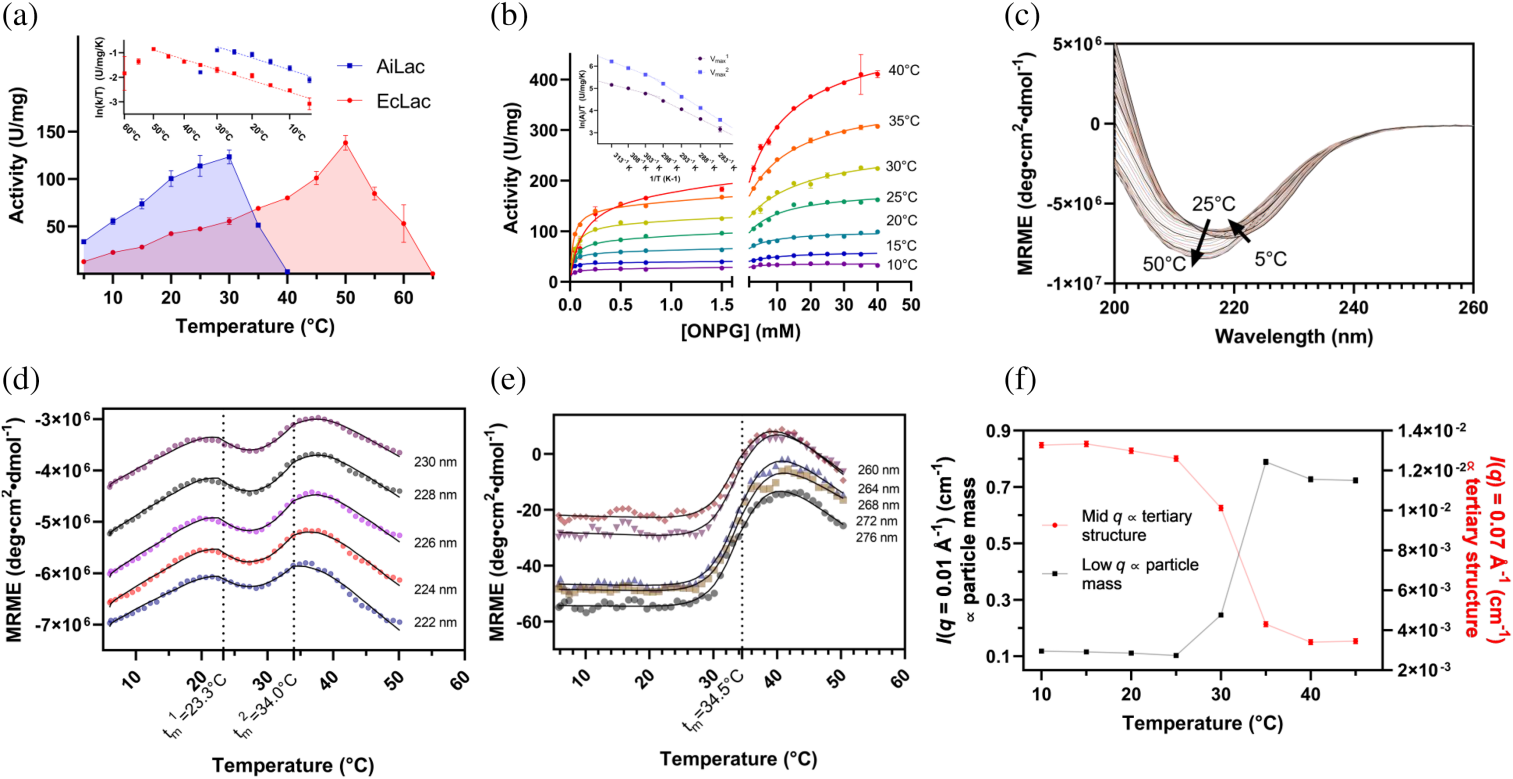
Effect of temperature on AiLac activity and structure. (a) Initial reaction rate of 8 nM AiLac (blue) and EcLac (red) in buffer A with 5 mM ONPG at different temperatures. The enzymes were pre‐equilibrated at the different temperatures for 10 min before measuring hydrolytic activity. (b) Michaelis–Menten plots of 8 nM AiLac in buffer A at different temperatures fitted to a two‐step binding scheme (Equation (1)). The enzyme was stored in ice right until the measurements; thus, we observe high rates above 30°C. The inset graph contains an Eyring plot ($\ln\left(\frac{k_{\mathrm{cat}}}{T}\right)=\frac{-∆H^{‡}}{R}\cdot \frac{1}{T}+\ln\frac{\kappa k_{B}}{h}+\frac{\Delta S^{‡}}{R}$) of maximal rates of both phases (*V*_max_^1^ and *V*_max_^2^) fitted to two linear regressions for 10–25°C and 30–40°C. (c) Thermal unfolding of 1.3 μM AiLac in buffer A measured by far‐UV CD at different temperatures with a 0.5°C/min gradient. Only data up to 50°C are shown, after which the protein aggregated, leading to strong light dispersion. (d) Data from panel (c) shown for selected wavelengths along the temperature gradient. The lines are the best fits to a two‐step transition using Global3 analysis software. (e) Thermal unfolding of 5 μM AiLac in buffer A measured by near‐UV CD at different temperatures with a 0.5°C/min gradient. As in (c), only data up to 50°C and selected wavelengths along the temperature gradient (shapes) are fitted with a single‐step transition using Global3 analysis software (black lines). (f) SAXS data from Figure S2b showing low‐*q* (proportional to mass) and mid‐*q* (proportional to tertiary structure) values as a function of temperature. All experiments are performed in PBS pH 7.0 with 0.1 mM Mg^2+^.

We next investigated how temperature‐induced changes in AiLac's activity and structure relate to each other. Activity experiments were performed at various temperatures, where both enzyme and substrate were pre‐incubated separately at a given temperature for 10 min before mixing and subsequently measuring initial velocity over a 10‐min period. This showed that AiLac and EcLac have temperature optima (*t*_opt_) of 30 and 50°C, respectively (Figure 2a). While the two proteins have essentially identical specific activity at their respective *t*_op_ (124 ± 7 and 138 ± 8 U/mg for AiLac and EcLac, respectively), AiLac showed ~2‐fold higher specific activity than EcLac toward ONPG at the AiLac *t*_opt_ of 30°C (but was completely inactive at the EcLac *t*_opt_ of 50°C). To obtain specific activation parameters, the initial specific activity *k* was used for an Eyring plot (ln (*k/T*) versus 1/*T*, cf., legend to Figure 2), which provides the enthalpy and entropy of activation (Figure 2a inset). The two enzymes have identical enthalpies of activation but different entropies (Table 1). While EcLac activity rises exponentially up to *t*_opt_, AiLac activity starts to level off above 20°C, suggesting unfolding events taking place already at 25°C (Figure 2a). To investigate this effect further, we performed Michaelis–Menten kinetics experiments with AiLac between 10 and 40°C. In this experiment, cooled enzyme is introduced to pre‐incubated substrate solution, and the initial velocity is measured for 1 min immediately after mixing, in contrast to the earlier experiment (Figure 1a) where the enzyme was pre‐ncubated for 10 min. This approach reduces the time available to the enzyme for thermal inactivation and therefore leads to observable activity at 40°C (Figure 2b). Here we observed biphasic kinetics, which yielded two individual Michaelis constants (*K*_m_^1^ and *K*_m_^2^) and their corresponding maximal velocities (*V* ^1^_max_ and *V* ^2^_max_) when fit by Equation (1) (Figure 2b). In an Eyring plot, both *V* ^1^_max_ and *V* ^2^_max_ show a linear dependency between 10 and 25°C (298 K), followed by a kink around 300 K and a new linear dependency with a lower slope as the temperature increases (Figure 2b, inset). The biphasic kinetics may represent the binding of multiple substrate molecules, further analysis of which is outside the focus of the present investigation.

**TABLE 1:** Experimental data for temperature‐dependent properties of AiLac and EcLac.

	AiLac	EcLac
*t*_opt_	30°C	50°C
Δ*H* ^‡^	8.03 ± 1.1 kcal/mol	7.84 ± 0.47 kcal/mol
Δ*S* ^‡^	25.0 ± 3.8 cal/mol	22.5 ± 1.6 cal/mol
*t*_m_ (far‐UV CD)	23.3 ± 0.1°C^a^, 34.0 ± 0.1°C^b^	53.6 ± 0.3°C
*t*_m_ (near‐UV CD)	34.5 ± 0.1°C	58.7 ± 0.4°C
Inflection point (DSF)	35 ± 0.9^c^–40.9 ± 0.1°C^d^	63.3 ± 0.2°C

^a^ First transition is calculated using Global 3 Thermal Global Analysis Software (Applied Photophysics, Leatherhead, UK) using a linear baseline for both the native and unfolded states.

^b^ Second transition is calculated using Global 3 Thermal Global Analysis Software (Applied Photophysics, Leatherhead, UK) using a linear baseline for both the native and unfolded states.

^c^ 3.3 nM protein concentration.

^d^ 33.9 μM protein concentration.

### 2.4 CD and SAXS show AiLac unfolds mainly around *t*_opt_

To investigate the process of thermal denaturation of AiLac in more detail, far‐UV CD spectra (260–200 nm) were recorded at steadily increasing temperatures (Figure 2c). Upon heating, the minimum of the CD spectrum shifts from ⁓ 222 nm toward ⁓ 215 nm, suggesting loss of *α*‐helical structures and prevalence of *β*‐sheets at high temperatures, which may be an indication of aggregation (Kelly et al., 2005). When plotted against temperature, ellipticities around 222–230 nm show two transitions which can be fitted to a double‐sigmoidal unfolding curve (Equation (4) described in “Materials and Methods”), giving a minor transition at 23.3°C and a major transition at 34.0°C (Figure 2d). When repeating this experiment in the near‐UV region (320–260 nm) we only see one transition with a *t*_m_ of 36.8°C (Figure 2e). Both far‐ and near‐UV CD show a *t*_m_ value consistent with a loss of activity due to unfolding slightly above the activity *t*_opt_ of 30°C (Figure 2a). This indicates that enzyme activity is lost upon global unfolding. The additional transition with *t*_m_ of 23.3°C seen by far‐UV CD leads to a more subtle change in activity, manifesting as a change in the enthalpy of activation.

In addition, thermal unfolding of AiLac was investigated by SAXS analysis, where an AiLac sample was heated from 10 to 45°C in steps of 5°C and a 30‐min measuring time (Figure S2b). Here, we observed a transition between 25 and 35°C where AiLac started to aggregate and form complexes with a larger mass (seen by an increase at low‐*q* values, Figure 2f) and the tertiary structure was broken down (seen by a decrease in mid‐*q* values, Figure 2f). This transition involved a shift in radius of gyration (*R*_g_) from 4.3 nm at 25°C to 7.4 nm at 30°C and 12.0 nm at 35°C (Table ^S1^). Gratifyingly, the *R*_g_ of 4.3 nm is close to the size of a dimeric structure (4.39 nm); however, the *R*_g_ of 12.6 nm is far from a fully unfolded monomer of ~60 nm (assuming *R*_g_ ~ *N* ^0.588^, where *N* = 1042 aa) (Flory, 1953). Both transitions for low‐ and mid‐*q* values have a midpoint around 32°C, in agreement with CD data above. However, DLS data recorded in parallel with DSF show that aggregation only starts above 40°C (Figure S1b), which is likely due to SAXS being run at 11.5 times higher protein concentration.

### 2.5 AiLac is unfolded at lower [Urea], and exhibits lower refolding capacity than EcLac

**FIGURE 3:**
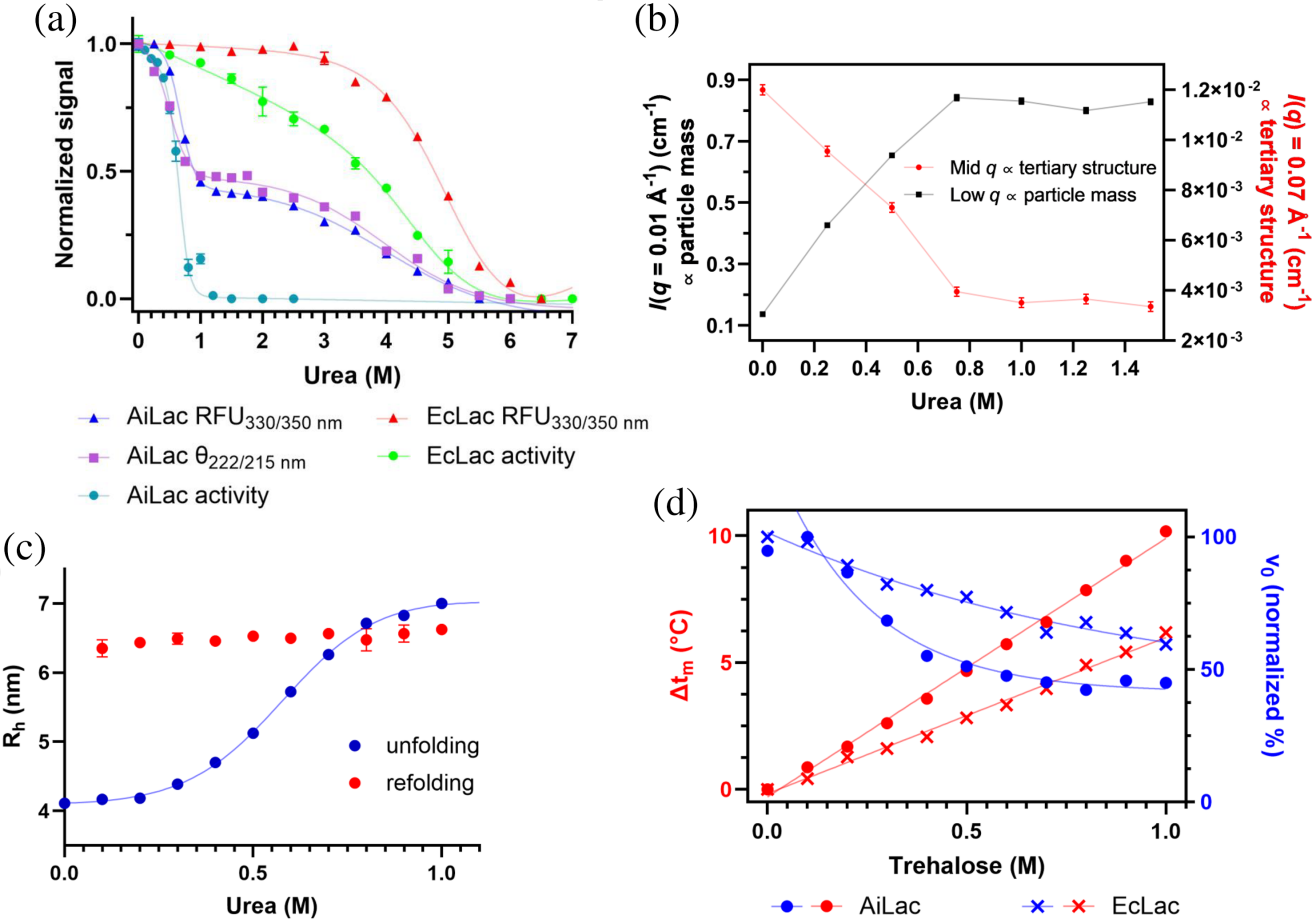
Stability and flexibility of AiLac. (a) Stability of AiLac and EcLac in urea at RT measured by changes in fluorescence (triangle), far‐UV ellipticity (square), and catalytic rate (circles). Protein concentration was 1.3 μM for far‐UV and fluorescence experiments and 8 nM for activity assays. Single‐step transitions are fitted to Equation (3), while two‐step transitions are fitted to Equation (4). (b) Values extracted from SAXS data in Figure S2c of AiLac showing low‐*q* (proportional to mass) and mid‐*q* (proportional to tertiary structure) as a function of urea concentration. (c) FIDA analysis of 0.1 μM AiLac showing *R*_h_ for unfolding and refolding in urea. (d) Change in melting temperature (Δ*t*_m_) of 0.8 μM AiLac (red circles) and EcLac (red crosses) induced by trehalose (left *y*‐axis). Catalytic rate of 8 nM AiLac (blue circles) and EcLac (blue crosses) in trehalose (right *y*‐axis). All experiments are performed in PBS pH 7.0 with 0.1 mM Mg^2+^.

We now compare the stability and activity of AiLac with that of EcLac in the presence of increasing amounts of the chemical denaturant urea. EcLac follows a single sigmoidal transition with reasonably coinciding midpoints at 5.0 M urea for activity (ONPG hydrolysis) and 5.1 M urea for tertiary structure (Trp fluorescence) (Figure 3a). There is a sloping baseline for activity below the major transition, which we attribute to a general inhibitory effect of urea prior to unfolding. In contrast, while AiLac activity is lost in a single transition with a midpoint at 0.66 M urea, Trp fluorescence and far‐UV CD measurements show a double transition with midpoints at ca. 0.6 M and ca. 4 M urea (Figure 3a). Thus, the initial transition is responsible for the complete loss of activity but only partial loss of secondary and tertiary structure.

The first transition of AiLac at low urea concentration was further investigated by SAXS and FIDA. SAXS revealed that unfolding and aggregation had started at the lowest measured value of 0.25 M urea, which was seen as an increase in particle mass (increase in intensity at low‐*q* values) and loss of tertiary structure (decrease in intensity at mid*‐q* values) compared to 0 M urea (Figure 3b and S2c). These changes plateau at 0.8 M urea, after which no significant transitions were observed. Furthermore, these two parameters changed in parallel, which indicated that AiLac was unfolded by urea, and these unfolded structures immediately formed larger aggregates. Similarly, FIDA performed at 0.1 μM AiLac showed an increase in the hydrodynamic radius *R*_h_ above 0.4 M urea, which reaches a plateau at 1.0 M urea (Figure 3c). Finally, the enzymes' ability to refold by dilution from the urea‐unfolded state of AiLac and EcLac was tested by intrinsic fluorescence and activity assays. Upon dilution of AiLac into 0.1 M urea from 1.1 M urea, only 15.1% ± 0.6% of the original fluorescence signal was regained (Figure S3a). Lack of refolding was confirmed by activity assays in which AiLac only recovered 1.9% ± 0.3% of its original activity in 0.1 M urea (Figure S3a). Similar results are observed for EcLac, where refolding of the protein from 6 M urea to 1 M urea resulted in 56% ± 0.7% fluorescence recovery, but only restoration of 3.8% ± 0.9% of the original activity (Figure S3b). These results showed that the unfolding of both enzymes was essentially irreversible, preventing determination of the free energy of unfolding. FIDA also revealed that the *R*_h_ of the urea‐unfolded state (~ 7 nm) only decreased to a very small extent upon dilution of urea and remained well above the value of 4.2 nm seen in the absence of urea (Figure 3c). This was further evidence that the early AiLac unfolding transition was irreversible.

### 2.6 AiLac is more affected than EcLac by flexibility‐reducing trehalose

To examine the role of structural flexibility in the activity of AiLac and EcLac, we turned to compounds known to reduce protein flexibility. Ammonium sulfate turned out to be inappropriate given its strong inhibitory effect on enzymatic activity (data not shown). Instead, we used trehalose, known to stabilize proteins *i.a*. by reducing flexibility (Jain & Roy, 2009), and measured its effect on both enzymes in terms of activity (ONPG hydrolysis) and stability (DSF). Trehalose decreased the activity of both AiLac and EcLac, but to different extents; at 0.5 M trehalose, AiLac and EcLac retained 50% and 75% activity, respectively (Figure 3d). In contrast, AiLac was stabilized by trehalose in terms of inflection point (IP) (corresponding to *t*_m_) with a factor of 10.2°C/M, which is 60% more than the 6.15°C/M of EcLac (Figure 3d). These results were consistent with the expectation that cold‐active enzymes are more reliant on flexibility and therefore more sensitive than mesophilic enzymes to the decrease in flexibility caused by stabilizers.

### 2.7 AlphaFold model predicts lack of subunit complementation

**FIGURE 4:**
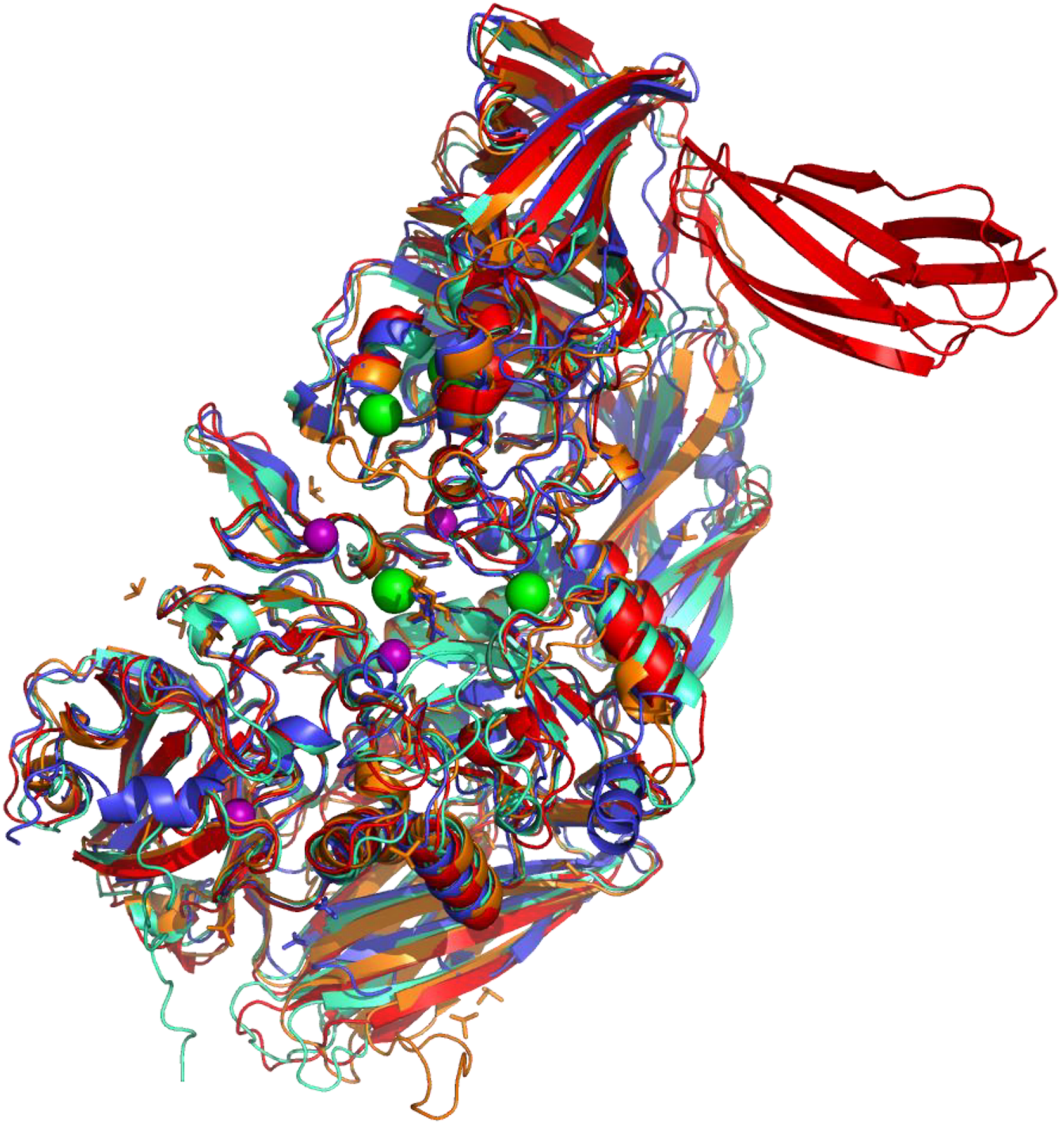
Structural model of AiLac showing a monomeric AF2 model (teal) aligned with monomer units of ArLac (6SEB, blue), EcLac (1JYN, orange), and TmLac (red, 6S6Z).

Having established the lower thermal and chemical stability of AiLac and its relatively higher flexibility compared to EcLac, we speculated whether these stability aspects were reflected in its structural features. AiLac's tendency to aggregate precluded direct structural analysis by x‐ray crystallography or cryoEM (data not shown). Instead, we turned to *in silico* modeling and utilized Alphafold 2.2 (AF2) to predict the hypothetical structure of an AiLac monomer unit. The resulting model fit well with monomer units of the structural homologs, the tetrameric EcLac (PDB entry 1JYN) (root‐mean square deviation, RMSD = 4.7 Å), the dimeric ArLac (PDB entry 6SEB) (RMSD = 7.1 Å), and the octameric TmLac (PDB entry 6S6Z) (RMSD = 3.6 Å) (Figure 4). The AF2 model of AiLac predicted a structure that was identical to domains 1–5 in EcLac. Interestingly, tetrameric EcLac has a loop (residues 272–288) that completes the active site of a neighboring subunit. The sequence corresponding to this loop was not found in AiLac. Rather, AiLac had an insertion of residues 434–443 predicted to form an internal loop extending over the active site (Figure S4). This change theoretically allows for the activity of single monomeric units of AiLac in contrast to EcLac, which is only active as a tetramer (Juers et al., 2012).

*De novo* prediction of oligomeric states of proteins is generally difficult (Fraser et al., 2016). Homology prediction tools such as Swiss‐Model (Waterhouse et al., 2018) simply transfer information of a homolog's quaternary state to the model, which leaves the resulting modeling scores as measures of prediction quality. When we modeled AiLac in Swiss‐Model against its homologs, we obtained similar QMEANDisCo scores (1JYN, 0.70 ± 0.05; 6SEB, 0.63 ± 0.05; 6S6Z, 0.74 ± 0.05), which estimated global model quality with the structures of the said homologs (Studer et al., 2020). As no individual model scored sufficiently higher than others, we decided to experimentally establish the quaternary structure of AiLac, that is, the number of subunits per protein complex.

### 2.8 Gel filtration and multiple‐angle light scattering suggest co‐existence of AiLac monomer with higher‐order species

**FIGURE 5:**
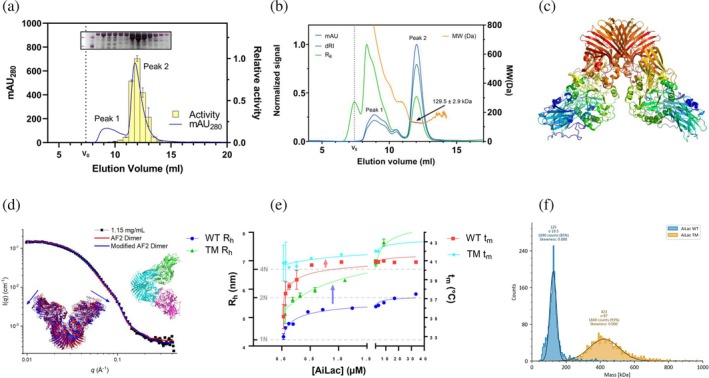
Oligomeric state and structure of AiLac. (a) Elution profile measured by UV absorption at 280 nm (left *y*‐axis) of 500 μL of 4 mg/mL AiLac loaded onto a Superdex 200 gel filtration column, divided into 0.5 mL fractions. Each fraction was tested for activity in technical triplicates using the photometric 5 mM ONPG assay in buffer A (right *y*‐axis). The full SDS‐PAGE of the fractions (a part of which is shown here as inset) can be found in Figure S5a. (b) Elution profile of 200 μg in 50 μL AiLac from a Superdex 200 gel filtration column connected to MALS, measuring absorption at 280 nm (mAU), refractive index change (dRI) and Raleigh ratio (*R*_θ_) (left *y*‐axis, normalized). This may be compared to the molecular size of eluted species (right *y‐*axis) deduced from light scattering. *V*_0_ is indicated by a stippled line. (c) Dimeric structure of AiLac predicted by AlphaFold 2.2 in the software package ParallelFold 1.1 with a set of AiLac sequences using “multimer_preset” setting. The resulting model is shown in cartoon with rainbow color spectrum from blue to red (N‐terminus to C‐terminus). (d) SAXS data of 1.15 mg/mL AiLac fitted with either the AF2 dimer (red) or the modified AF2 dimer from simulation 6 optimized by Monte Carlo simulations (blue). The structure for each of these structures is shown in the same color as the fit. The blue arrows indicate that the blue modified structure is slightly more open than the red unmodified structure. The right inset shows the three parts of the dimer (green, cyan, and magenta) that were allowed to move relative to each other during the Monte Carlo simulations. (e) The effect of protein concentration on self‐association and stability. 125 nM fluorescently labelled WT AiLac (blue circles) or 60 nM TM AiLac (green triangles) was titrated against unlabelled enzyme (WT or TM, respectively) in buffer A measured by FIDA (left *y*‐axis). Thermal stability of AiLac at different protein concentrations was measured by DSF, represented as melting temperature *t*_m_ (right *y*‐axis) and shown by red squares for WT AiLac and light blue diamonds for TM AiLac. Both data sets are fitted to Equation (5). All experiments are performed in PBS pH 7.0 with 0.1 mM Mg^2+^. The semi‐transparent arrows indicate shifts in *R*_h_ or *t*_m_ values due to mutagenesis. (f) Mass photometric analysis of the MW of WT and TM AiLac. Clearly, TM has undergone a significant increase in mass, consistent with tetramer formation.

Size exclusion chromatography (SEC) in the last protein purification step showed that AiLac gave rise to two overlapping peaks eluting at 8–11 and 11–14 mL, respectively (Figure 5a). Both peaks contained a ~120 kDa protein according to SDS‐PAGE (Figure 5a insert, whole gel in Figure S5a), which is close to the theoretical 119.13 kDa of monomeric AiLac, but activity was only associated with the second peak. In contrast, both the major EcLac fractions showed activity (Figure S5b). Based on elution volumes of standardized globular proteins (Figure S5c), the second peak of AiLac corresponded to 128 kDa ± 1.3, in close agreement with the protein's monomer MW of 119 kDa. The first peak of AiLac suggested a MW of 325 ± 1.3 kDa based on elution volume. We used two approaches to obtain more exact molecular weights. Firstly, we applied SEC in combination with MALS, which confirmed the MW of the second peak to be 129.5 ± 2.9 kDa (Figure 5c). In this SEC setup, the first peak was slightly better resolved into multiple overlapping peaks due to a lower injection volume (50 μL rather than 500 μL used in Figure 5a). The peak around 8–10 mL consisted of a heterogeneous mixture of different species >400 kDa, suggesting aggregation, while two shoulders eluting around 10–11.5 mL had sizes of 400–500 and ~ 270 kDa, respectively, corresponding to tetramer and dimer structures, respectively. Second, we applied SAXS measurements on each of the two peaks obtained in Figure 5a (Figure S5d). This led to a mass of 1994 ± 200 kDa for the first peak, corresponding to 17 ± 2 monomers, which was most likely a combination of various complexes. The second peak gave a mass of 211 ± 21 kDa corresponding to 1.7 ± 0.2 monomers. The SAXS curve for the first peak showed a significant decrease in intensity at mid‐*q* range was significantly decreased compared to the second peak (Figure S5d), suggesting loss of tertiary structure. This is consistent with a scenario in which the first peak contains a combination of aggregates and other large complexes, as also observed in MALS intensity of scattered light (*R*_*θ*_) data (green line, Figure 5b). This scenario also explains the peak's lack of enzymatic activity (Figure 5a). The large differences in mass obtained in SAXS compared to the other techniques may be a time effect, as our SAXS measurements each take 0.5 h.

### 2.9 SAXS data can be fitted by the predicted AF2 dimeric structure

SAXS data for AiLac's second peak showed a mass corresponding to 1.7 ± 0.2 monomers, that is, close to a dimer. When AF2 is asked to predict a dimer structure, this leads to an arrangement where monomers interact along a single interface in their C‐termini (Figure 5c). We calculated the expected scattering of the AF2 dimer model and fitted it to the measured data, varying only the concentration, a parameter describing the hydration around the protein, and a constant background. We obtained a reasonable fit with a reduced χ^2^ value of 13.8 and a concentration of 1.046 ± 0.009 mg/mL, which was close to the expected concentration of 1.15 mg/mL (red line in Figure 5d, summarized in Table S2). This suggests that the AF2 dimer model is a good representation of the solution structure of AiLac. While the fit was visually good, small deviations were seen for the entire *q* range (Figure 5d). To resolve this, the dimeric structure was split into three parts that were allowed to move relative to each other during a Monte Carlo optimization that also includes distance restraints along the backbone and excluded volume penalties (three parts shown in green, cyan, and magenta in the right inset in Figure 5d). We justified this on the basis that they are connected by loop regions, which should allow them to move relative to one another in solution. The connections are between V626 and S327 for both of the constituting monomers. The connections of the two monomers were kept as predicted by Alphafold2. Ten individual Monte Carlo simulations were performed to optimize the structure against the measured SAXS data. For these ten simulations, an average χ^2^ value of 2.89 ± 0.10 was obtained (Table S2 shows values for individual simulations). This was a significant improvement, although the resulting structures only had an average RMSD of 3.0 ± 0.5 Å compared to the original structure (RMSD values for the individual structures are shown in Table S2). An example of the best fit (simulation 6) is seen in Figure 5d as the blue line calculated from the blue structure (left inset in Figure 5d). The blue and red structures are overlaid in the left inset of Figure 5d, which shows that they are very similar. However, the blue modified structure is slightly more open than the red structure, as indicated by the blue arrows.

### 2.10 AiLac exists in a monomer‐dimer equilibrium

The SAXS, SEC, and SEC‐MALS experiments were performed at relatively high (1–3 mg/mL) protein concentrations, which might promote oligomerization. To probe dimerization over a wider concentration range, we used flow‐induced dispersion analysis, FIDA, which provides the *R*_h_ of fluorescently labelled protein and can be performed at concentrations down to a few μg/mL. To monitor transitions between different states over a 1000‐fold range in concentrations, we titrated fluorescently labeled AiLac with increasing amounts of non‐labeled AiLac (blue data points in Figure 5e). *R*_h_ of the labeled protein increased from 4.3 ± 0.1 nm at 32 nM (4 μg/mL) to 5.8 ± 0.03 nm at 33.5 μM (4 mg/mL). Gratifyingly, the FIDA PDB size predictor program predicted *R*_h_ to be 4.2 and 5.7 nm for monomeric and dimeric AiLac, respectively, using the SAXS‐optimized AF2 structure. EcLac *R*_h_ was measured to be 6.5 ± 0.1 nm, which corresponded well with a predicted value of 6.7 nm. Our AiLac data could be fitted with a monomer‐dimer association model (Equation (5)), leading to a dissociation constant (*K*_D_) of 0.23 ± 0.20 μM (blue line in Figure 5e). Having established that AiLac forms a homodimer in a concentration‐dependent manner, we investigated whether the quaternary structure affected the stability of the enzyme. To do this, we utilized DSF at various enzyme concentrations. Plotting melting temperatures (*t*_m_) of DSF unfolding curves revealed that a high concentration of AiLac indeed increased AiLac's thermal stability (red data points in Figure 5e). Going from 33 nM to 33.5 μM increases *t*_m_ by 5.7°C. Fitting the stability data to Equation (5) gave a *K*_D_^app^ of 0.15 ± 0.09 μM (red line in Figure 5e), in good agreement with FIDA data.

### 2.11 Highly evolved fitness mutations at the interface promote oligomeric assembly and thermostability

Protein sequences, evolved over billions of years, harbor extensive information about structural and functional constraints. Evolution‐based machine learning models, trained on individual protein families, have shown promising results in predicting protein structure from residue co‐variation (Jumper et al., 2021; Morcos et al., 2011) and designing novel sequences that are stable and functional (Russ et al., 2020; Tian et al., 2018; Tian et al., 2022). These models are also effective in predicting single mutational effects predicted on protein sequence fitness (Hopf et al., 2017). However, the extent to which evolutionary information captured by these models can be used to design proteins with higher‐order oligomerization remains unclear. We hypothesized that most GH2 *β*‐galactosidases are more stable than AiLac due to stable oligomeric conformations such as tetramers. By capturing the evolutionary pattern of AiLac homologs, we aimed to identify mutations that could shift the oligomerization preference toward dimer and tetramer, thereby enhancing the stability. Deep learning methods are very powerful in learning high‐dimensional data distributions (Lopez et al., 2018; Rezende et al., 2014). In this study, we constructed an evolutionary model of the GH2 *β*‐galactosidase family using a Bayesian Variational Autoencoder model called EVE (Frazer et al., 2021). We sought to determine whether higher‐order oligomeric assembly could be induced through evolutionarily favorable mutations identified by the model at the dimeric interface. Table 2 lists the top 11 single mutations based on the evolutionary fitness score suggested by the model. We focus on the top three candidates, namely V648E, F702L, and T742V. While the last two mutations are conservative amino acid replacements, the mutation V548E represents a more significant change. We hypothesize that this substitution may stabilize quaternary interactions since Val648 is located at the dimer interface, and replacing it with Glu could introduce hydrogen bonds or salt bridges with nearby residues on the adjacent subunit, strengthening dimer/tetramer interactions. We produced both single mutant constructs and a triple mutant (TM) using USER cloning site‐directed mutagenesis to investigate their impact on the structure and stability of AiLac. Upon gel filtration of all four constructs, we observed a significant shift in the distribution of the oligomeric states as seen by the comparatively higher intensity of the initial elution peak (Figure S7). In contrast to the WT AiLac, both peaks contained active enzyme, suggesting that the higher‐order species of the mutant fold into active structures. We then utilized FIDA to investigate whether we could measure the oligomeric state of the higher‐order assembly observed by SEC, and for this, we chose to focus on the TM variant of AiLac. Attempts to confirm the oligomeric assembly using SAXS were unsuccessful due to aggregation (data not shown). Instead, we used two complementary approaches to investigate quaternary structure, namely mass photometry (MP) and FIDA. MP uses interferometric scattering (which scales with mass) to estimate the MW of individual molecules (Young et al., 2018). Application to TM reveals a remarkably clear shift in mass from ~120 to ~420 kDa, consistent with tetramerization (Figure 5f). For FIDA, we titrated unlabeled protein to a small amount of labeled protein and measured the *R*_h_ (blue line, Figure 5e). Gratifyingly, we observed that the AiLac TM has a *R*_h_ of a dimer already at the lowest measured concentration, which assembles into a tetramer as the concentration approaches 10 μM. Furthermore, we also measured the thermal stability of AiLac TM across the titration series and once again observed a trend of increasing *t*_m_ as a function of protein concentration (orange line, Figure 5e). AiLac TM has a significantly higher *t*_m_ of 40.8 ± 1.9°C at 32.8 nM, which increases to 42.0 ± 0.1°C at 4.2 μM. Furthermore, similar increased thermal stabilities are seen for all three single mutants as well (Figure S8), which aligns well with the observed peak shifts in the gel filtration experiments (Figure S7). Overall, our experiments confirmed that these high evolutionary fitness mutations successfully shift the propensity toward tetramerization and improve evolutionary fitness while maintaining functionality. These findings can serve as a general protein engineering principle for modulating protein oligomerization.

**TABLE 2:** Prediction of stabilizing mutations at the dimeric interface of AiLac obtained by the evolutionary model EVE (Frazer et al., 2021).

Mutation	Evolutionary fitness^a^
V648E	−2.53
F702L	−1.97
T742V	−1.08
T745P	−0.94
V744A	−0.87
R748E	−0.21
K743A	0.47
L967S	1.75
L740V	2.62
P741K	3.77
P968S	4.16

^a^ Lower value means higher fitness.

## 3 DISCUSSION

The motivation for our study of the AiLac *β*‐galactosidase was to understand how the stability and structure of AiLac influence activity at low temperature. We discovered that AiLac associates with a highly unstable dimer, whose formation is protein concentration dependent. Furthermore, the dimerization enhances the thermal stability of AiLac. We were able to induce further oligomerization by inducing stabilizing mutations at the dimer interface, which led to the formation of AiLac in a dimer‐tetramer equilibrium and further increased the thermal stability of the protein.

### 3.1 Cold activity of AiLac

AiLac has a temperature optimum at 30°C and 2–3 times higher specific activity toward ONPG than EcLac at 5–30°C, which makes AiLac a classic example of a cold‐active enzyme (Cavicchioli & Siddiqui, 2006; Feller & Gerday, 2003; Fields & Somero, 1998). The biphasic aspect of the kinetics might arise from a heterotrophic active site that is activated at high substrate concentrations (Tracy & Hummel, 2004). Far‐UV and near‐UV CD thermal denaturation experiments showed major unfolding events at *t*_m_ = 34°C or *t*_m_ = 34.7°C, respectively. However, far‐UV also showed a slight loss of 222 nm signal at *t*_m_ = 23.3°C, which indicates an early rearrangement involving *α*‐helical structures. This fits well with *V*_max_ dependency on temperature, where we observed a change of slope at temperatures above 25°C. These two observations are likely connected, as a deviation from linearity between ln(*k*_cat_)/*T* and *T* ^−1^ is likely caused by either thermal denaturation or a temperature‐dependent reversible inactivation. An alternative explanation is a non‐linear difference in heat capacity, Δ*Cp* ^‡^, signifying temperature‐dependent change dynamics of protein rearrangement during catalysis, for example, lid opening (Nguyen et al., 2017). However, the changes in far‐UV CD spectra around 23°C suggest local structural rearrangements as a simpler explanation. The local nature of this change is supported by the fact that global changes observed by near‐UV CD, FIDA, and SAXS all occur ≥30°C. Loss of activity at lower temperatures than that of global unfolding is one of the characteristic traits of psychrophilic enzymes (Feller & Gerday, 2003). It is unclear whether this is also the case for AiLac; certainly, there is no major loss in activity at this temperature, but more subtle changes may affect the otherwise steep rise in activity with temperature in this interval.

### 3.2 Highly unstable and flexible structure of AiLac

Psychrophilic enzymes tend to be less stable than their mesophilic and thermophilic homologs due to an increase in structural flexibility (Feller & Gerday, 1997). A superoxide dismutase from the psychrophilic *Pseudoalteromonas haloplanktis* has a midpoint of denaturation of 4.2 M urea compared to 8.2 M urea of its mesophilic homolog from *E. coli* (Merlino et al., 2010). A psychrophilic *α*‐amylase from *P. haloplanktis* has a midpoint of transition at 0.9 M guanidine, whereas the mesophilic counterpart from pig pancreas has its midpoint at 2.6 M guanidine (Feller et al., 1999) (note that guanidine is a stronger denaturant than urea, typically being twice as efficient (Lim et al., 2009; Rashid et al., 2005)). Even by the standards of unstable psychrophilic enzymes, AiLac is remarkably sensitive to perturbation. Urea titration measured by aromatic fluorescence shows that the psychrophilic enzyme unfolds by two transitions. The first, involving loss of function with a [urea]^50%^ of 0.67 M, is dramatically lower than the 5 M midpoint for unfolding and loss of function of EcLac. The initial AiLac transition involves an increase in particle size, no matter whether measured at high (SAXS) or low (FIDA) protein concentrations, suggesting that cooperative unfolding is independent of quaternary structure. A 119 kDa protein would have an *R*_h_ of 11.6 nm and *R*_g_ of 12.6 nm, assuming a fully unfolded state (Wilkins et al., 1999). However, FIDA data showed an *R*_h_ transition to a ⁓7 nm particle at 1 M urea, which is nevertheless larger than a folded monomer (*R*_h_ = 4.4 nm). SAXS measurements showed a transition from an *R*_g_ value of 5.2 nm in buffer to 11.9 nm when unfolded in urea and 4.4–12 nm when heated from 20 to 35°C (Table ^S1^). Utilizing the FIDA size predictor, which can predict both *R*_h_ and *R*_g_, a monomer would have an *R*_g_ of 3.3 nm, while the dimer is predicted to be 4.4 nm. The latter corresponds to the initial size of AiLac in the SAXS experiments, suggesting that a dimeric structure is maintained before unfolding. Therefore, the data indicate that the transition is to an unfolded species, no matter whether a monomer or a multimer is the starting point. Furthermore, unfolding through this transition is irreversible in terms of both activity and stability, as only approximately 2% of activity is observed upon refolding, 15% of the fluorescence shift signal is restored, and no decrease in *R*_h_ was observed using FIDA upon refolding. The connection between stability and flexibility is important, as sensitivity toward perturbants is not necessarily an adaptive feature reflecting high flexibility. Therefore, we assessed the effect of trehalose on AiLac and compared it to EcLac. Trehalose is an organic osmolyte whose stabilizing effect has been linked to a decrease in structural flexibility of proteins (Fedorov et al., 2011). Indeed, we saw that AiLac is significantly more affected by trehalose than EcLac, both in terms of loss of activity and stability. This strongly suggests that AiLac has a more flexible structure at room temperature (RT) than EcLac and that its high activity is connected to its instability at elevated temperatures.

### 3.3 Oligomerization

SEC analysis combined with MALS indicated that the AiLac enzyme is a functional monomer. However, SAXS experiments combined with the AlphaFold model presented an elongated dimer as the most likely structure for AiLac. Although the initial protein concentration was similar in both experiments, we suspected that dilution of AiLac across the gel filtration column caused monomerization of the protein. The discrepancy between SEC‐MALS and SAXS was solved using FIDA, which showed that the monomers form dimers in a concentration‐dependent manner with a *K*_D_ = 0.3 μM (36 μg/mL). This observation means that while the enzyme functions as a monomer under activity assessment conditions (typically 8 nM), we will mainly observe a dimeric structure at concentrations needed for SAXS (typically 8 μM). Furthermore, DSF measurements of AiLac at different concentrations showed that dimerization increases enzyme stability, which is important for the interpretation of experiments performed under various conditions. Interestingly, isothermal unfolding with urea measured by SAXS does not show increased stability compared to FIDA measurements, despite expected dimerization and its effect on stability. This discrepancy may be explained by differences in the experimental set‐ups and aggregation caused by high protein concentration. Few studies reporting on protein structure investigate concentration‐dependent oligomerization. Therefore, it is difficult to estimate the true oligomeric state of an enzyme at assay conditions unless it is specifically investigated.

There is high diversity in oligomeric formation of structural homologs to AiLac. The closest structural homologs include the dimeric *β*‐galactosidase from *Arthrobacter* sp. 32cB (Rutkiewicz et al., 2019), the tetrameric EcLac from *E. coli* (Matthews, 2005), and the octameric *β*‐galactosidase from the thermophilic *Thermotoga maritima* (Míguez Amil et al., 2020). Since AiLac shows the highest amino acid sequence similarity to enzymes with the homotetrameric structure of EcLac, we expected AiLac to form a tetramer. EcLac is exclusively functional in its fully assembled tetrameric state, as the active site is completed by a loop extending from the neighboring subunit (Juers et al., 2012). This same loop is not conserved in AiLac, and therefore, it is not expected that multimerization would be required for enzymatic activity. Low conservation in this loop region is not unique to AiLac, however. Neither of the structural homologs present this type of intermolecular active site completion, and both the thermo‐ and psychrophilic *β*‐galactosidases have a more solvent‐exposed binding site than EcLac. Interestingly, the AlphaFold model predicted another loop occupying the same space but one from the same subunit (residues 508–518 in AiLac amino acid numeration).

It is noteworthy that the AiLac dimer resembles one half of the EcLac tetramer interacting across the activating interface. This is different from the dimeric structure of the *β*‐galactosidase from *Arthrobacter* sp. 32cB (ArLac), which has a “head‐to‐tail” interaction across the monomers with the active site facing toward the interface (Rutkiewicz et al., 2019). A structural study of ArLac revealed that its unique dimeric arrangement minimizes buried surface area (8.9%), which represents a large fraction of solvent‐exposed amino acid area of a multimer compared to a monomer. This is similar to the psychrophilic homologs (14.7%) and is significantly lower than the meso‐ and thermophilic homologs (22.4%–32.5%). This observation was linked to cold adaptation. Note that even lesser buried surface area is observed for our dimeric AiLac model (3.3%), suggesting that cold adaptation of the AiLac enzyme involves minimization of buried surface area.

Our findings of induced multimerization in the single and the triple mutants predicted by an evolutionary model show that very few additional interactions between the monomeric units are necessary to stabilize the dimer structure and extend it to a tetrameric state, as demonstrated by mass photometry, which in turn also equipped the enzyme with increased thermal stability. This shows not only that our protein design method is highly efficient at identifying these kinds of mutations but also that changes in quaternary structure are evolutionarily highly accessible through a small number of mutations. We also note that according to both the AlphaFold‐based dimer model and the SAXS data, the three mutations (V648E, F702L, and T742V) are clustered at or near the subunit‐subunit interface rather than near the catalytic cleft. In AiLac, the active site is largely formed by intramolecular loops (notably residues 434–443, predicted to occupy the substrate‐binding region within the same chain). Because none of the three engineered substitutions lie in or near the substrate‐binding groove, they do not directly perturb the substrate‐binding loops or catalytic residues. This spatial separation helps explain why the enzyme retains activity even though the mutants significantly alter oligomerization behavior. Moreover, unlike the tetrameric E. coli *β*‐galactosidase (where part of the active site is formed by a neighboring subunit's loop), AiLac's monomer appears to carry its own complete catalytic machinery. Thus, engineering additional contacts at the dimer interface can improve stability without disturbing the essential geometry of the catalytic site.

### 3.4 The controversial role of de‐multimerization as an adaptation to cold

Oligomeric state, stability, and activity of AiLac are all highly dependent on pH. AiLac showed the highest activity in acidic solutions; however, at 20°C it aggregated below neutral pH and formed amorphous multimers above neutral pH. Altogether, these data showed that AiLac experiences a tight balance between activity and stability in regard to activity with a narrow window of function around neutral pH. Under these conditions, we have shown that the enzyme forms a dimer that dissociates into monomers at low concentrations. Oligomerization into higher‐order assemblies as a mechanism of thermostabilization is well established, although in most cases it has been proposed mainly through indirect evidence based on thermally stable, high‐order proteins from thermophilic bacteria and archaea (Míguez Amil et al., 2020; Robinson‐Rechavi et al., 2006; Walden et al., 2001). Conceptually, this makes good sense since oligomerization makes unfolding more difficult (and therefore stabilizes the protein) because both inter‐ and intramolecular contacts need to be broken. Besides this, there is additional evidence. For example, SDS resistance has been linked to oligomerization (Manning & Colón, 2004), and artificially oligomerized proteins, either through engineering or *de novo* evolution, have been shown to result in increased stability (Fraser et al., 2016; Kirsten Frank et al., 2002; Kuhlman et al., 2001). We also demonstrate that AiLac dimerization at higher protein concentration increases thermal stability. Whether de‐oligomerization of enzymes is an active adaptation toward efficient catalysis at low temperatures or a passive loss of high‐temperature activity remains controversial. In a wider scope, all six isolated *A. ikkensis* relatives, which also produce *β*‐galactosidases, happen to be mesophilic bacteria with halophilic tendencies, but with no psychrophiles among them (Table S3). In four of these, the *β*‐galactosidase genes and their respective flanking sequences are syntenic (i.e., close in the bacterial genome) to that of AiLac (Figure S6), showing the conservation of the whole cluster. The high identity of the AiLac sequence and its respective cluster collinearity among the relatives indicates that *A. ikkensis* is a mesophilic bacterium that has adapted toward survival in the cold environment of the Ikaite columns relatively recently in its evolutionary history. This is supported by the fact that the Greenlandic ice sheet covered the Ikka Fjord until ca. 8000 years ago, and thus, the maximal age of the ikaite columns is approximately 8000 years (Buchardt et al., 2001). Furthermore, we have previously shown that the majority of bacteria isolated from ikaite columns are eurypsychrophilic (activity spanning a broad range of temperatures) and not stenopsychrophilic (narrow‐range activity) (Schmidt et al., 2006; Stougaard et al., 2002). Nonetheless, our work suggests a link between low stability, high flexibility, and high activity at low temperatures of AiLac and its relation to the unique dimer arrangement that dissociates to monomers at low enzyme concentrations, which we were able to modify to form a tetramer through a triple mutation that has high evolutionary fitness at the dimerization interface.

## 4 MATERIALS AND METHODS

Note that lower‐case *t* signifies temperature in Celsius and upper‐case *T* represents absolute temperature in Kelvin.

### 4.1 Materials

All chemicals and enzymes were purchased from Merck unless stated otherwise. The following buffers were used: buffer A (phosphate buffer saline +0.1 mM MgCl_2_): 2.7 mM KCl, 8 mM Na_2_HPO_4_, 2 mM KH_2_PO_4_, 137 mM NaCl, 0.1 mM MgCl_2_ pH 7.0; buffer B (binding): buffer A + 10 mM imidazole; buffer C (washing); buffer A + 40 mM imidazole; buffer D (elution): buffer A + 400 mM imidazole; buffer E (circular dichroism): 10 mM sodium phosphate, 0.1 mM MgCl_2_, pH 7.0.

### 4.2 AiLac production

*E. coli* BL21(DE3) cells were transformed with a pET9a(USER) plasmid carrying the gene encoding AiLac (EC# 3.2.1.23; GenBank ACO52514.1). Recombinant cells were plated on LB agar containing 50 μg/mL kanamycin (Km50), incubated overnight at 37°C, and suspended in 20 mL LB Km50 medium. The pre‐culture was used to inoculate 1 L LB Km50 and incubated at 37°C, 150 rpm. When the culture reached OD_600_ = 0.8, expression was induced with 0.1 mM Isopropyl ß‐d‐1‐thiogalactopyranoside and 1 mM rhamnose, and the temperature was reduced to 18°C. After 4 h of additional growth, the cells were harvested by centrifugation at 4000 *g* for 30 min in 1 L flasks at 4°C in a Sorvall Lynx 6000 centrifuge (Thermo). The cell pellet was resuspended in 10 mL binding buffer (buffer B) and the cells were lysed by 10 min of sonication using a 2 mm Microtip probe mounted to a Q500 sonicator (Qsonica, Connecticut, USA) set to 20 s pulse/pause intervals at 20% intensity. The lysate was centrifuged at 15,000 *g* for 20 min at 4°C in 50 mL tubes, and the supernatant was kept at −20°C before purification. Thawed lysate was filtered through a 0.45 μm syringe filter and loaded onto 2 mL Ni^2+^‐charged Ni‐NTA beads in a 15 mL plastic column, followed by 15 min of incubation on a rotating table at 4°C. The lysate was eluted, and the beads were washed with 5 column volumes of buffer C. The protein was eluted with 2 column volumes of buffer D, and the eluate was immediately exchanged to buffer A using disposable PD‐10 columns (Cytiva, Marlborough, USA) following standard gravity protocol. Purity was assessed by SDS‐PAGE, and the protein was stored at −80°C.

### 4.3 Gel filtration

Further purification and size estimation were performed by SEC using a Superdex 200 Inc. column (Cytiva, Marlborough, USA) connected to an Äkta Pure 25 M system (Cytiva, Marlborough, USA). The flow was set to 0.5 mL/min, and 0.5 mL of 3 mg/mL protein solution was injected. The protein eluted at 8–14 mL and was collected in 0.5 mL fractions. Protein concentration, purity, and activity of each fraction were assessed by absorption at 280 nm, SDS‐PAGE, and ONPG activity assays, respectively. Proteins for size comparison purchased from Sigma in lyophilized form— hen egg white lysozyme (HEWL), carbonic anhydrase (CA), bovine serum albumin (BSA), and EcLac— were dissolved in PBS pH 7.0 to a 1 mg/mL concentration and run on SEC with the same column and settings as AiLac.

### 4.4 Photometric activity assay

Activity of the AiLac and EcLac (purchased from Sigma) was assessed by hydrolysis of ONPG. The experiments were performed either utilizing a single cell set‐up with a Lambda 25 UV/VIS spectrophotometer connected to PTP‐1 Peltier system (Perkin Elmer, Massachusetts, USA) using 10 mm quartz cuvettes (Hellma, Switzerland) or a plate‐reader set‐up with Varioscan LUX microplate reader (Fisher, Massachusetts, USA) using Nunc 96‐well Clear Plates (Fisher, Massachusetts, USA). The reactions were initiated by adding 0.2 mg/mL enzyme stock, yielding 1 μg/mL (8 nM) final concentration, and were followed by absorption reading at 420 nm. The reaction rate is expressed in μmol product per minute per mg enzyme (U/mg) using an extinction coefficient of hydrolyzed ONPG at the relevant pH, ONP‐ being the light‐absorbing species with ε_ONP‐_ = 4600 M^−1^ cm^−1^ and pKa = 7.2, giving the pH dependence $\varepsilon_{\mathrm{pH}}=\varepsilon_{\mathrm{ONP}-}∙f_{\mathrm{ON}P^{-}}=\varepsilon_{\mathrm{ONP}-}∙\frac{10^{\mathrm{pH}-\mathrm{pKa}}}{1+10^{\mathrm{pH}-\mathrm{pKa}}}$ (= 1741 M^−1^ cm^−1^ at pH 7.0).

Biphasic kinetics are modeled according to the sequential binding scheme:

$$E+2S\overset{K_{1}}{\Leftrightarrow }E:S+S\overset{K_{2}}{\Leftrightarrow }E:S_{2}$$

Here, *E* is the enzyme, *S* is the substrate, and *K*_1_ and *K*_2_ are the equilibrium dissociation constants for the first and second binding step. This leads to the following equation:

$$\begin{array}{l} K_{1}=\frac{\left[E\right]\left[S\right]}{\left[\mathrm{ES}\right]};K_{2}=\frac{\left[\mathrm{ES}\right]\left[S\right]}{\left[ES_{2}\right]};\\ {\left[E\right]}_{0}=\left[E\right]+\left[\mathrm{ES}\right]+\left[ES_{2}\right]↔\left[\mathrm{ES}\right]=\frac{{\left[E\right]}_{0}}{1+\frac{K_{1}}{\left[S\right]}+\frac{\left[S\right]}{K_{2}}}\Leftrightarrow \end{array}$$

(1) $$\begin{array}{rcl} k_{\mathrm{obs}}^{\mathrm{cat}} & = & \frac{V}{{\left[E\right]}_{0}}=\frac{k_{1}^{\mathrm{cat}}∙\left[\mathrm{ES}\right]+k_{2}^{\mathrm{cat}}∙\left[ES_{2}\right]}{{\left[E\right]}_{0}}\\ & = & \frac{\left[\mathrm{ES}\right]\left(k_{1}^{\mathrm{cat}}+k_{2}^{\mathrm{cat}}∙\frac{\left[S\right]}{K_{2}}\right)}{{\left[E\right]}_{0}}=\frac{k_{1}^{\mathrm{cat}}+k_{2}^{\mathrm{cat}}∙\frac{\left[S\right]}{K_{2}}}{1+\frac{K_{1}}{\left[S\right]}+\frac{\left[S\right]}{K_{2}}} \end{array}$$

Magnesium dependency titration data is fitted with the following equation, adapted from Mogensen et al., (2005):

(2) $$k_{\mathrm{cat}}^{\mathrm{obs}}=\frac{\frac{k_{\mathrm{cat}}^{a}\cdot K_{1}}{\left[Mg^{2+}\right]}+k_{\mathrm{cat}}^{b}+\frac{k_{\mathrm{cat}}^{c}\cdot \left[Mg^{2+}\right]\;}{K_{2}}}{\frac{K_{A}}{\left[Mg^{2+}\right]}+1+\frac{\left[Mg^{2+}\right]}{K_{B}}}$$

where $k_{\mathrm{cat}}^{a}$, $k_{\mathrm{cat}}^{b}$, and $k_{\mathrm{cat}}^{c}$ are rate constants with zero, one, and two Mg^2+^ ions bound, respectively. *K*_A_ and *K*_B_ are dissociation constants for the binding of the first and second Mg^2+^ ions, respectively.

### 4.5 Thermal stability measured by circular dichroism

Thermal stability was assessed on a Chirascan‐plus CD spectropolarimeter (Applied Photophysics, Leatherhead, UK) in combination with a TC1 Temperature controller (Quantum Northwest, Liberty Lake, USA). Far‐UV and near‐UV experiments were performed at 190–260 nm and 250–320 nm, respectively. For far‐UV experiments, 300 μL of protein at 0.2 mg/mL (unless stated otherwise in Results section) was transferred to a 1 mm quartz cuvette (Hellma, Switzerland), while near‐UV experiments were performed using 1 mL of 0.5 mg/mL (unless stated otherwise in “Results” section) in a 5 mm quartz cuvette (Hellma, Switzerland). In both experiments, the CD was set to 1 nm bandwidth, 0.5 s time per point, single repeat, and overwrite set temperature with temperature measured in the cuvette. The unfolding data were fitted using Global 3 Thermal Global Analysis Software (Applied Photophysics, Leatherhead, UK) using a linear baseline for both native and unfolded states.

### 4.6 Thermal stability measured by DSF

Thermal stability of AiLac and EcLac in trehalose was assessed with a Tycho NT.6 (Nanotemper, München, Germany) using the ratio of emission at 330 and 350 nm (excitation at 280 nm) and a pre‐set scanning rate of 30°C/min at 80% laser power. The protein concentration was 0.15 mg/mL in buffer A. Prior to these assays, SEC‐purified protein in buffer A was incubated in 0–1 M trehalose for 4 h at RT.

Thermal stability of AiLac at different protein concentrations was performed on Nanotemper Panta (Nanotemper, München, Germany) using the ratio of emission at 330 and 350 nm (excitation at 280 nm) at 7% laser power and a scanning rate of 0.5°C/min. SEC‐purified AiLac was measured at concentrations between 4 mg/mL and 3.9 μg/mL. Thermal stability was assessed using built‐in software by calculating the mid‐point of denaturation as the IP seen as a peak in the plot of the first derivative of the signal versus temperature.

### 4.7 Isothermal stability measured by chemical denaturation

Protein stability was assessed by fluorescence and CD after incubation in various urea (UltraPure) concentrations. Trp fluorescence was measured with a Varioscan LUX microplate reader (Fisher, Massachusetts, USA) at RT using Nunc 96‐well Black Bottom Plates (Fisher, Massachusetts, USA). Samples (150 μL in triplicates) contained 0.2 mg/mL protein and were incubated for 6 h at RT to achieve equilibrium. They were excited at 280 nm while emission was measured from 310 to 450 nm using a 5 nm bandwidth and 100 ms per point. For a two‐state (sigmoidal) transition, the resulting data are fitted to the following equation (Fersht et al., 1999):

(3) $$\text{Signal}=\frac{\left(\alpha_{N}+\beta_{N}\left[\text{urea}\right]\right)+\left(\alpha_{D}+\beta_{D}\left[\text{urea}\right]\right)10^{\frac{m_{D-N}\left(\left[\text{urea}\right]-{\left[\text{urea}\right]}^{50\%}\right)}{-\mathrm{RT}}}}{1+10^{\frac{m_{D-N}\left(\left[\text{urea}\right]-{\left[\text{urea}\right]}^{50\%}\right)}{-\mathrm{RT}}}\;}$$

*α*_N_ and *α*_D_ are baseline signals for the native and the denatured states, respectively, at 0 M urea, whereas *β*_N_ and *β*_D_ are the respective baseline slopes. [urea]^50%^ is the urea concentration at which *K*_D‐N_ = [D]/[N] = 1. *m*_D‐N_ describes the linear relationship between log *K*_D‐N_ and [urea]. In the case of a three‐state transition (double sigmoidal), the following equation was used:

(4) $$\text{Signal}=\frac{\left(\alpha_{N}+\beta_{N}\text{[urea]}\right)+\left(\alpha_{I}+\beta_{I}\text{[urea]}\right)10^{\frac{m_{I-N}\left(\text{[urea]}-{\text{[urea]}}_{\left(I\right)}^{50\%}\right)}{-\mathrm{RT}}}+\left(\alpha_{D}+\beta_{D}\text{[urea]}\right)10^{\frac{m_{D-I}\left(\text{[urea]}-{\text{[urea]}}_{\left(D\right)}^{50\%}\right)}{-\mathrm{RT}}}}{1+10^{\frac{m_{I-N}\left(\text{[urea]}-{\text{[urea]}}_{\left(I\right)}^{50\%}\right)}{-\mathrm{RT}}}+10^{\frac{m_{D-I}\left(\text{[urea]}-{\text{[urea]}}_{\left(D\right)}^{50\%}\right)}{-\mathrm{RT}}}}.$$

This equation is an expanded version of Equation (1) with added intermediate signal baseline (*α*_I_) and baseline slope (*β*_I_), together with [urea]^50%^_(I)_ where *K*_I‐N_ = [I]/[N] = 1 and [den]^50%^_(D)_ where *K*_D‐I_ = [D]/[I] = 1. *m*_I‐N_ and *m*_D‐I_ describe the linear relationships between log *K*_I‐N_ and log *K*_D‐N_, respectively, and [urea].

### 4.8 Thermal and isothermal structure and stability by SAXS

SAXS data were obtained on NanoSTAR from Bruker AXS (Karlsruhe, Germany) at Aarhus University. The instrument is flux optimized with a liquid gallium metal jet x‐ray source from excillum (Kista, Sweden) and homebuilt scatterless slits. More information on the optimized instrument can be found here (Lyngso & Pedersen, 2021). Background subtraction and conversion to absolute scale using a measured water sample at 20°C was carried out using the SUPERSAXS program software package (Oliveira, C.L.P. and Pedersen, J.S., unpublished). Data are plotted as a function of *q*, which is the modulus of the scattering vector. It is defined as $q=4\sin\left(\theta \right)/\lambda$, where 2*θ* is the angle between the incident and scattered beam, and λ is the wavelength, which is $\lambda_{\mathrm{Ga}}=1.34\;Å$ for this instrument. All samples were measured for 1800 s at 20°C using buffer A unless otherwise stated. To determine the pH dependency, samples of 0.40 mg/mL AiLac were prepared with pH ranging from 6.0 to 9.5. pH was adjusted by titration of HCl or NaOH to 50 mM bis‐tris propane. However, only samples from pH 7.0 to 9.5 were measured, as samples at pH 6.0 and 6.5 showed large visible aggregates. The mass was determined by $M=I\left(0\right)\times N_{A}/\left(c\times \Delta \rho_{m}^{2}\right)$, where *I*(0) is the intensity extrapolated to *q* = 0, *N*_A_ is Avogadro's number, *c* is the protein concentration is mg/mL, and $\Delta \rho_{m}$ is the scattering contrast per mass. For a typical protein, this can be estimated to $2.0\times 10^{10}$ cm/g. Mass determinations done by SAXS have an uncertainty of around 10%, as they rely on the precision of the concentration determination, the uncertainty of estimating *I*(0), and how well the protein in question fits with standard value for the scattering contrast per mass used in the equation. SAXS data were also obtained for both peaks from the SEC purification. The first peak has a concentration of 0.85 mg/mL and the second peak a concentration of 1.15 mg/mL. For the modeling of the dimer structure, we first compared the scattering of the Alphafold predicted dimer to data. This was done by calculating the theoretical scattering curve using the program wlsq_pdbx (Steiner et al., 2018). This program fits the concentration, hydration contribution, and a constant background.

To further optimize the structure, it was divided into three parts separated by loop regions. These three parts could move relative to each other when performing ten individual Monte Carlo optimizations, each with 100 steps. The program includes soft distance restraints along the backbone between the parts as well as excluded volume penalties. When running the simulations, the program automatically calculates an expected concentration, which can be done as the data are on an absolute scale. After the simulations, each structure was aligned to the Alphafold dimer and a root mean square deviation (RMSD) value was calculated. Values from the simulations are given either on an individual basis or as an average of the ten simulations, with the standard deviation listed as the uncertainty. Urea dependence of AiLac structure was investigated by measuring AiLac with a concentration of 1.2 mg/mL at increasing urea concentrations. The sizes in the form of radius of gyration (*R*_g_) were obtained using the model‐independent indirect Fourier transformation developed by Glatter (1977). Temperature dependence on AiLac structure was investigated by increasing the temperature gradually for a sample of 1.10 mg/mL AiLac. The temperature was raised by 5°C and the sample was equilibrated for 150 s before the next 1800 s measurement started.

### 4.9 Protein model by AlphaFold

The structure of full sequence AiLac (GenBank/EMBL/DDBJ accession no. FJ811841) was predicted using a modified version of DeepMind's AlphaFold 2.2 (AF2) (Jumper et al., 2021) ParallelFold v1.1 (ParaFold) (Zhong et al., 2022). The monomeric structure was modelled using “monomer_preset” while the dimeric structure was modelled by selecting “multimer_preset” with two AiLac sequences in succession.

### 4.10 Oligomeric state and stability measured by FIDA

200 μg of AiLac in buffer A purified by SEC was fluorescently tagged with Alexa Fluor 488 NHS Ester (Succinimidyl Ester) (Invitrogen, Massachusetts, USA) by adding 10% V/V 1 M sodium bicarbonate and 2.4 μL of 7.2 mM Alexa 488 stock (1:3 protein to label molar ratio), followed by 3 h incubation in the dark at 20°C on a rolling table. Free label was removed by desalting on a disposable PD MidiTrap G‐25 column (Cytiva, Marlborough, USA) following the standard gravity protocol. FIDA was performed using a FIDA 1 platform (Fida Biosystems, Søborg, Denmark). Here, 125 nM (WT AiLac) or 60 nM (TM AiLac) labeled protein was utilized as an indicator molecule with various amounts (0–1 mg/mL) of non‐labeled AiLac used as analyte. 62.5 and 31.5 nM measurements of WT AiLac were performed by serial dilution of the 125 nM labeled sample. The labeled AiLac was pre‐mixed with unlabeled AiLac at different concentrations and was incubated at 4°C for 4 h before starting the experiment. The resulting elution curve was fitted to a two‐species model using standard fitting (75% of curve length) with one hydrodynamic radius (*R*_h_) locked to 0.6 nm, representing unbound label. The resulting *R*_h_ values calculated in the FIDA software were fit to the following equations:

(5) $$\begin{array}{c} R_{h}=R_{h^{\text{mono}}}+\frac{2\left[D\right]}{\left[M_{0}\right]}\left(R_{h^{\text{dimer}}}-R_{h^{\text{monomer}}}\right)\; \end{array}$$

where $R_{h^{\text{monomer}}}$ and $R_{h^{\text{dimer}}}$ are sizes from monomer and dimer, respectively. [*M*_0_] is the initial monomer concentration, while [*D*] is dimer concentration obtained by solving a quadratic equation for the equilibrium:

(6) $$\begin{array}{c} \left[D\right]=\frac{\left(4\left[M_{0}\right]+K_{D}\right)\pm \sqrt{{\left(4M_{0}+K_{D}\right)}^{2}-16M_{0}^{2}}}{8}, \end{array}$$

where *K*_D_ is the dissociation constant of the dimer into themonomer.

### 4.11 Oligomeric state measured by SEC‐MALS

A Superdex 200 10/300 GL Inc. gel filtration column (Cytiva, Marlborough, USA) was connected to a 1260 Infinity II Liquid Chromatography System (Agilent, USA). The light scattering signal was detected downstream of the column by a DAWN8+ MALS detector (Wyatt, Dernbach, Germany) (λ = 658 nm); the refraction index (RI) was measured by an Optilab T‐rEX RI detector (Wyatt, Dernbach, Germany) (λ = 658 nm) and UV absorbance was measured by the UV detector (λ = 280 nm) built into the liquid chromatography system. Hvad med R(theta) som du også henviser til? The experiments were performed at room temperature. The system was pre‐equilibrated with buffer A at a constant 1 mL/min flow, and the detectors were calibrated with 20 μL 2 mg/mL bovine serum albumin. Ni‐NTA purified protein was thawed and spun at 13,000 rpm for aggregate removal, and 50 μL of 1.5 mg/mL protein was loaded by the auto sampler. The sample was loaded and eluted at a constant 0.5 mL/min flow in injection mode. The results were processed in Astra 7.1.1 software (Wyatt, Dernbach, Germany).

### 4.12 Interface stabilizing mutation prediction by evolution‐based deep learning model

We built an evolution‐based model for the AiLac protein homologs using a Bayesian Variational Autoencoder called EVE (Frazer et al., 2021). Specifically, for this deep neural network, both the encoder and decoder were configured with three layers. The hidden layer sizes for the encoder were set to 2000, 1000, and 300, respectively, while those for the decoder were set to 300, 1000, and 2000, respectively. A total of 40,000 epochs were used for the training process. This model learns the probabilistic distribution over 8544 natural sequences extracted from the UniRef100 database (Suzek et al., 2007). The protein homolog search method JackHMMER was employed with parameters set to an *E*‐value of 10^−4^ and eight iterations. Sequences with more than 25% gaps were discarded, (Johnson et al., 2010) and sequences within 95% sequence identity were removed from the training set.

Using the model, we derived the “evolutionary fitness score” for each amino acid variant, which estimates the relative likelihood of the variant compared to the wild type, by sampling from the approximate posterior distribution of the VAE. We focused on mutations at the intermolecular interface of the AiLac dimer structure, which was obtained by fitting to our SAX data. A pair of residues is defined as being in contact if any heavy atoms of any either residue were within 5.5 Å.

### 4.13 Site‐directed PCR mutagenesis USER Cloning

Mutagenesis of the WT AiLac was performed directly on the pET9a(USER) plasmid containing the AiLac sequence utilizing Site‐Directed PCR Mutagenesis USER Cloning (Geu‐Flores et al., 2007). The primers for the three single mutations (V648E, F702L, T742V) were designed using the AMUSER software (Genee et al., 2015). The TM was created by three consecutive rounds of mutagenesis using V648E, F702L, T742V primers, respectively. The plasmid was modified using PCR with the following conditions: an initial denaturation at 98°C for 30 s, followed by 30 cycles of 98°C for 19 s, 67°C (dependent on primer melting temperature) for 30 s, and 72°C for 3 min, with a final extension at 72°C for 3 min. The PCR mixture consisted of 19 μL Milli‐Q water, 2.5 μL forward primer, 2.5 μL reverse primer, 1 μL template DNA (approximately 50 ng/μL plasmid), and 25 μL PhusionU polymerase. The PCR product was purified using the GeneJET PCR Purification Kit (Thermo Fisher Scientific) and purified DNA was then treated with USER enzyme to create sticky ends by incubating a reaction mixture containing 1 μL 10× Cutsmart buffer, 8 μL DNA, 0.5 μL DpnI, and 0.5 μL USER enzyme at 37°C for 1 h. The modified plasmid DNA was chemically transformed into XL1‐Blue cells. Transformation involved thawing chemically competent XL1‐Blue cells on ice, adding 5 μL of the reaction mixture to the cells, and incubating on ice for 20 min. The cells were then heat‐shocked at 42°C for 2 min, followed by incubation with 0.5 mL LB at 37°C for 1 h. Transformed cells were plated on kanamycin agar plates, and 4 colonies were picked for plasmid purification and subsequent sequencing, which was performed using the Mix2Seq service by Eurofins Genomics. Plasmid DNA (5 μL at 50–100 ng/μL) was mixed with 5 μL of 1X sequencing primer provided in the Mix2Seq kit, and the samples were sequenced by Eurofins Genomics.

### 4.14 Mass photometry

The molecular mass of the proteins was measured on a TwoMP mass photometer (Refeyn, Oxford, England) at RT (21°C). Microscope slides (24 × 50 mm, 170 ± 5 μm, No. 1.5H, Paul Marienfeld, Germany) were cleaned sequentially by water bath sonication with Milli‐Q water, isopropanol, and Milli‐Q water again, then dried using a clean nitrogen stream. Eight‐well reusable silicone gaskets (CultureWell™ 50–3 mm DIA × 1 mm depth, 3–10 μL, Grace Bio‐Labs, Oregon, USA) were cut and assembled at the center of the cover slide. The system was calibrated by separately measuring the ratiometric contrast of BSA (66 kDa monomer, 132 kDa dimer) and thyroglobulin (670 kDa) at a final concentration of 20 nM. Calibration was performed by placing an 18 μL droplet of buffer in a well to enable focusing on the glass surface, followed by the addition of 2 μL of protein. To minimize the dissociation of molecular assemblies of AiLac, a higher dilution factor was used. Focusing was achieved using 19.75 μL of standard buffer, to which 0.25 μL of protein was directly added from a 1 mg/mL (8.4 μM) stock solution. This resulted in a final protein concentration of approximately 100 nM. DiscoverMP software (Refeyn Ltd., Oxford, UK) was used to generate distribution histograms, with average masses, peak width, and counts determined by Gaussian fitting at half‐height integration.

## AUTHOR CONTRIBUTIONS

**Jan S. Nowak:** Conceptualization; methodology; investigation; formal analysis; writing – original draft; writing – review and editing. **Nikoline Kruuse:** Methodology; conceptualization; formal analysis; investigation; writing – review and editing. **Helena Ø. Rasmussen:** Methodology; formal analysis; investigation; writing – review and editing. **Pengfei Tian:** Methodology; investigation; formal analysis. **Julie Astono:** Formal analysis; investigation. **Søren Schultz‐Nielsen:** Formal analysis; investigation. **Mariane S. Thøgersen:** Conceptualization; supervision; writing – review and editing. **Peter Stougaard:** Conceptualization; supervision; writing – review and editing. **Jan Skov Pedersen:** Formal analysis; supervision; writing – review and editing. **Daniel E. Otzen:** Conceptualization; methodology; formal analysis; supervision; funding acquisition; project administration; writing – original draft; writing – review and editing.

## CONFLICT OF INTEREST STATEMENT

The authors declare no conflict of interest.

## Supporting information


**Supplementary Figure S1:** Thermal stability of AiLac in 2 mM EDTA (a), 0.1 mM MgCl_2_ (b), 6.4 mM MgCl_2_ (c), and 102.4 mM (d) measured by fluorescence shift (left secondary axis) and scattering counts (right secondary axis) representing tertiary structure and on‐set of aggregation, respectively. Figure (e) shows scattering curves for all MgCl_2_ concentrations measured, where data beyond reaching the plateau is excluded due to the aggregates falling to bottom of the capillary, as seen in (c) and (d). The curves are fit to sigmoidal equation $S=S_{\min}+{\left(\frac{S_{\max}-S_{\min}}{1+(\frac{A_{50}}{\left[\mathrm{MgC}l_{2}\right]}}\right)}^{S_{f}}$, where *S* is the measured signal, *S*_min_ is the background signal, *S*_max_ is the plateau signal, *S*_*f*_ is the slope factor, and *A*_50_ is the midpoint of transition. In (f) we show the midpoint of aggregation (*A*_50_) as function of [MgCl_2_].
**Supplementary Figure S2:** 1800 s SAXS measurements of 1.1 mg/mL AiLac in various conditions: (a) pH ranging from 7.0 to 9.5 buffered by 50 mM bis‐tris propane, (b) in 10–45°C temperature range, and (c) chemical unfolding by 0.25–1.5 M urea.
**Supplementary Figure S3:** (a) Refolding of AiLac upon dilution of protein dissolved in 1.1 urea measured by fluorescence (blue) and enzymatic activity (purple). (b) Refolding of EcLac upon dilution of protein dissolved in 6 urea measured by fluorescence (red) and enzymatic activity (orange). Both the activity and fluorescence shift (350/330 nm) data are normalized to signal prior to denaturation (0 M urea).
**Supplementary Figure S4:** Overlay of the active site of the EcLac structure (1JYN) bound to lactose (beige) and the AF2 predicted structure of AiLac. Here, the active site of a single subunit EcLac is shown in orange, where a loop of neighboring subunit (red) completes the active site. AiLac AF2‐predicted structure is shown in teal, where the completing loop is found within the same subunit as the active site. The single Mg^2+^ ion in EcLac is shown in green and Na^+^ ions are shown in purple.
**Supplementary Figure S5:** (a) Full SDS‐PAGE of AiLac fractions from size exclusion chromatography found in Figure 5a showing AcLac bands at approx. 120 kDa. (b) Size exclusion chromatography (Superdex 200 Inc) of EcLac fractionated into 1 mL aliquots, which were tested for activity (shown in yellow columns) normalized to the highest activity within the dataset. (c) SEC of AiLac (black) compared to proteins with known molecular size: EcLac (orange), bovine serum albumin (BSA, blue), carbonic anhydrase (CA, red), and hen egg white lysozyme (HEWL, green). The inset shows the molecular weight of the proteins as a function of their elution volumes, where the linear regression is used for calculation of molecular weight of AiLac peaks. *V*_o_ in (b) and (c) signifies void volume of the column equal to 7.4 mL. (d) SAXS measurements of first and second peaks from the SEC purification in Figure 5a with concentrations indicated in brackets.
**Figure S6:** Comparative synteny analysis of *β*‐galactosidase (AiLac) gene clusters in *A. ikkensis* and its six isolated mesophilic relatives. Despite the mesophilic nature and halophilic tendencies of these relatives (as shown in Table S3), no psychrophiles were identified among them. The synteny of *β*‐galactosidase genes and their flanking sequences in four of these relatives mirrors the gene arrangement seen in *A. ikkensis*, demonstrating a conservation of the entire gene cluster.
**Figure S7:** Size exclusion profiles of AiLac and its mutants fractionated into 1 mL aliquotes, each being tested for activity using colorimetric enzyme assay: (a) overlay of all profiles normalized to the highest fluorescence within a dataset, (b) AiLac WT elution profile, (c) AiLac V648E, (d) AiLac F702L, (e) AiLac T742V, and (d) triple mutant AiLac.
**Figure S8:** Differential scanning fluorimetry measurement of AiLac WT (blue), V648E (red), F702L (green), T742V (purple), and triple mutant (orange) at different protein concentrations.

**Table S1:** *R*_*g*_ values obtained from SAXS data for AiLac at different temperatures (left columns, SAXS data in Figure S2b) and [Urea] (right column, SAXS data in Figure S2c)^a^.
**Table S2:** Fit values for AF2 dimer to measured SAXS data along with the individual and average results from the Monte Carlo simulations. *ρ*hydration describes the contribution from the hydration layer and can have a value between 0.2 and 1.3 depending on the protein.
**Table S3:** Overview of the six isolated *A. ikkensis* relatives that also produce *β*‐galactosidases.

## Data Availability

The data that support the findings of this study are available from the corresponding author upon reasonable request.
