# Supplementary material for: Quaternary stabilization of a GH2 β‐galactosidase from the psychrophile A. ikkensis, a flexible and unstable dimeric enzyme
Source: Protein Sci. 2025 Apr 25;34(5):e70141. doi: 10.1002/pro.70141 (PMC12023411; doi:10.1002/pro.70141)
Supplement: Supplementary file 1 — Supplementary Figure S1: Thermal stability of AiLac in 2 mM EDTA (a), 0.1 mM MgCl2 (b), 6.4 mM MgCl2 (c), and 102.4 mM (d) measured by fluorescence shift (left secondary axis) and scattering counts (right secondary axis) representing tertiary structure and on‐set of aggregation, respectively. Figure (e) shows scattering curves for all MgCl2 concentrations measured, where data beyond reaching the plateau is excluded due to the aggregates falling to bottom of the capillary, as seen in (c) and (d). The curves are fit to sigmoidal equation S=Smin+Smax−Smin1+(A50MgCl2Sf, where S is the measured signal, S min is the background signal, S max is the plateau signal, S f is the slope factor, and A 50 is the midpoint of transition. In (f) we show the midpoint of aggregation (A 50) as function of [MgCl2]. Supplementary Figure S2: 1800 s SAXS measurements of 1.1 mg/mL AiLac in various conditions: (a) pH ranging from 7.0 to 9.5 buffered by 50 mM bis‐tris propane, (b) in 10–45°C temperature range, and (c) chemical unfolding by 0.25–1.5 M urea. Supplementary Figure S3: (a) Refolding of AiLac upon dilution of protein dissolved in 1.1 urea measured by fluorescence (blue) and enzymatic activity (purple). (b) Refolding of EcLac upon dilution of protein dissolved in 6 urea measured by fluorescence (red) and enzymatic activity (orange). Both the activity and fluorescence shift (350/330 nm) data are normalized to signal prior to denaturation (0 M urea). Supplementary Figure S4: Overlay of the active site of the EcLac structure (1JYN) bound to lactose (beige) and the AF2 predicted structure of AiLac. Here, the active site of a single subunit EcLac is shown in orange, where a loop of neighboring subunit (red) completes the active site. AiLac AF2‐predicted structure is shown in teal, where the completing loop is found within the same subunit as the active site. The single Mg2+ ion in EcLac is shown in green and Na+ ions are shown in purple. Supplementary Figure S5: (a) Full SDS‐PAGE of AiLac [file PRO-34-e70141-s002.pptx]

## Slide 1
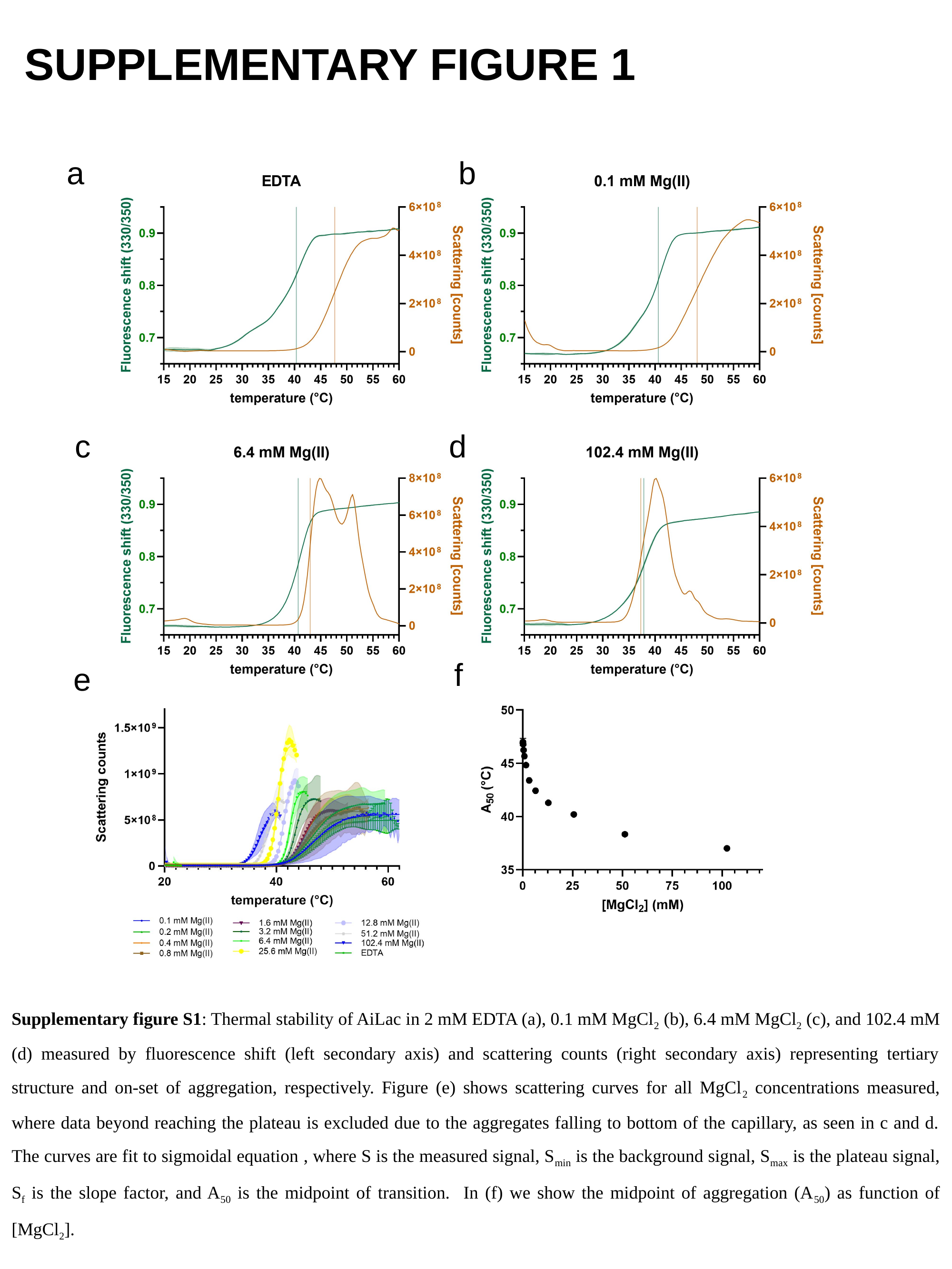

# Supplementary figure 1
a
b
c
d
f
e
07/04/202530/05/2022

## Slide 2
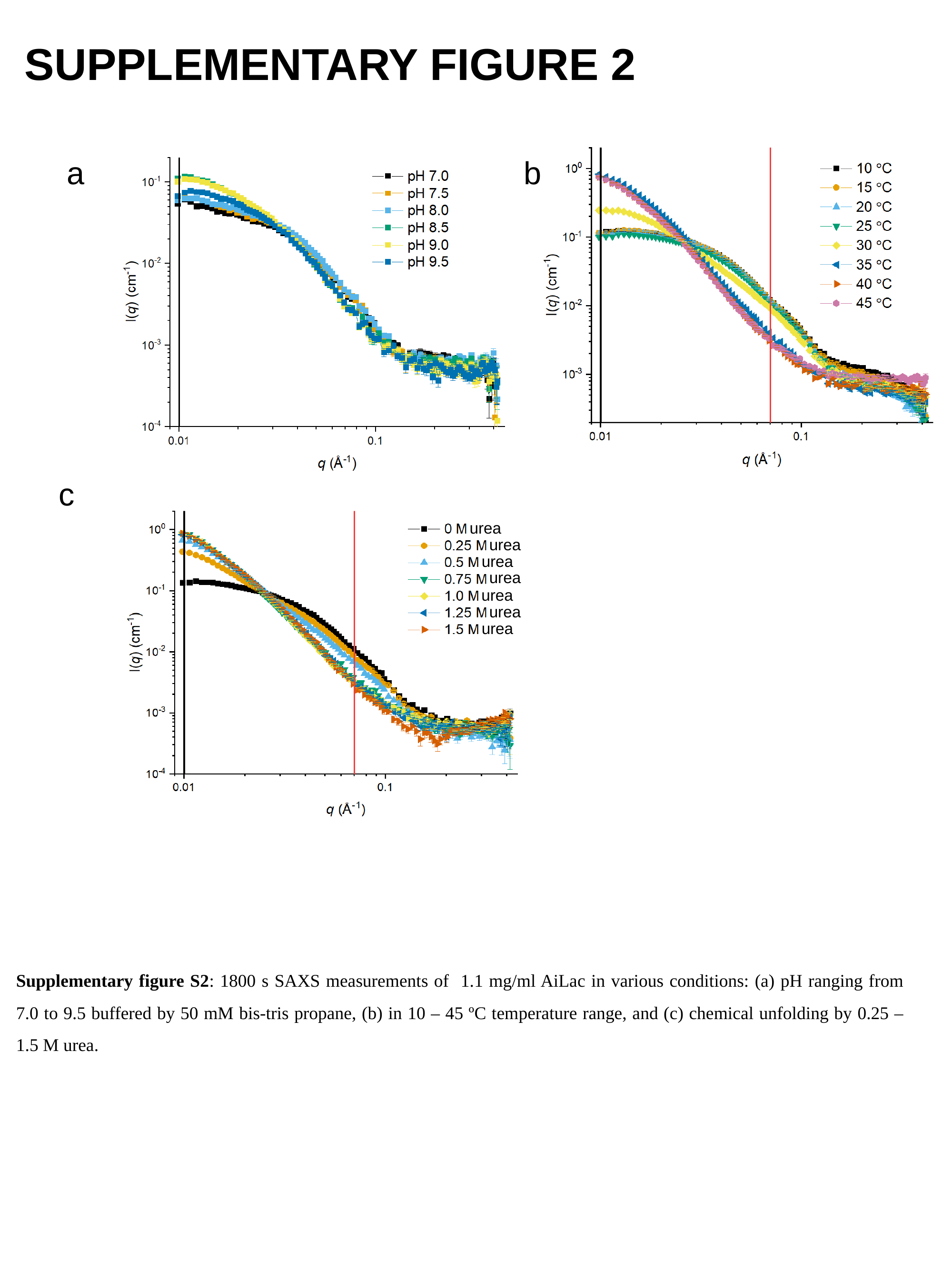

# Supplementary figure 2
a
b
c
urea
urea
urea
urea
urea
urea
urea
Supplementary figure S2: 1800 s SAXS measurements of 1.1 mg/ml AiLac in various conditions: (a) pH ranging from 7.0 to 9.5 buffered by 50 mM bis-tris propane, (b) in 10 – 45 ºC temperature range, and (c) chemical unfolding by 0.25 – 1.5 M urea.
07/04/202530/05/2022

## Slide 3
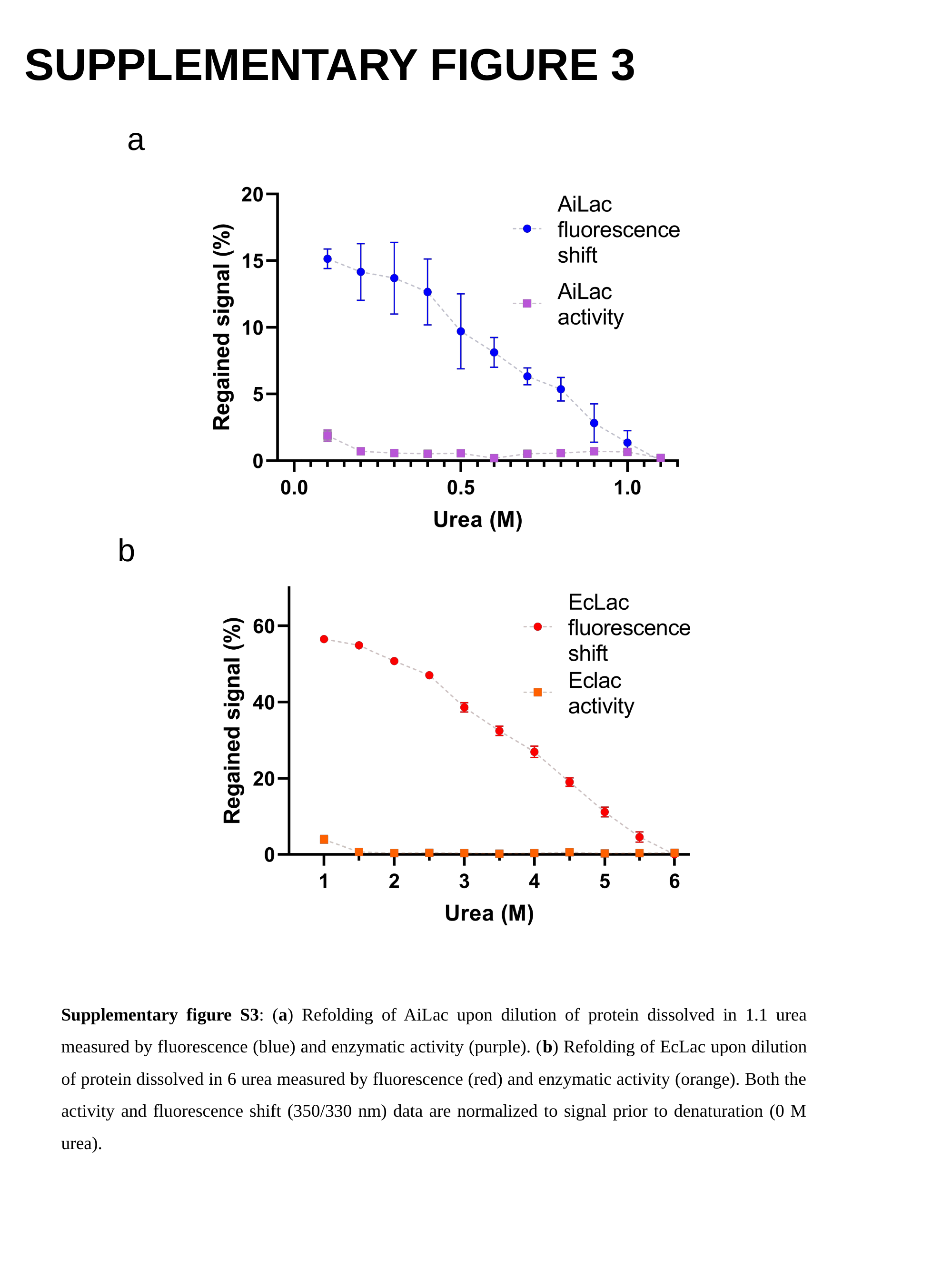

# Supplementary figure 3
a
b
Supplementary figure S3: (a) Refolding of AiLac upon dilution of protein dissolved in 1.1 urea measured by fluorescence (blue) and enzymatic activity (purple). (b) Refolding of EcLac upon dilution of protein dissolved in 6 urea measured by fluorescence (red) and enzymatic activity (orange). Both the activity and fluorescence shift (350/330 nm) data are normalized to signal prior to denaturation (0 M urea).
07/04/202530/05/2022

## Slide 4
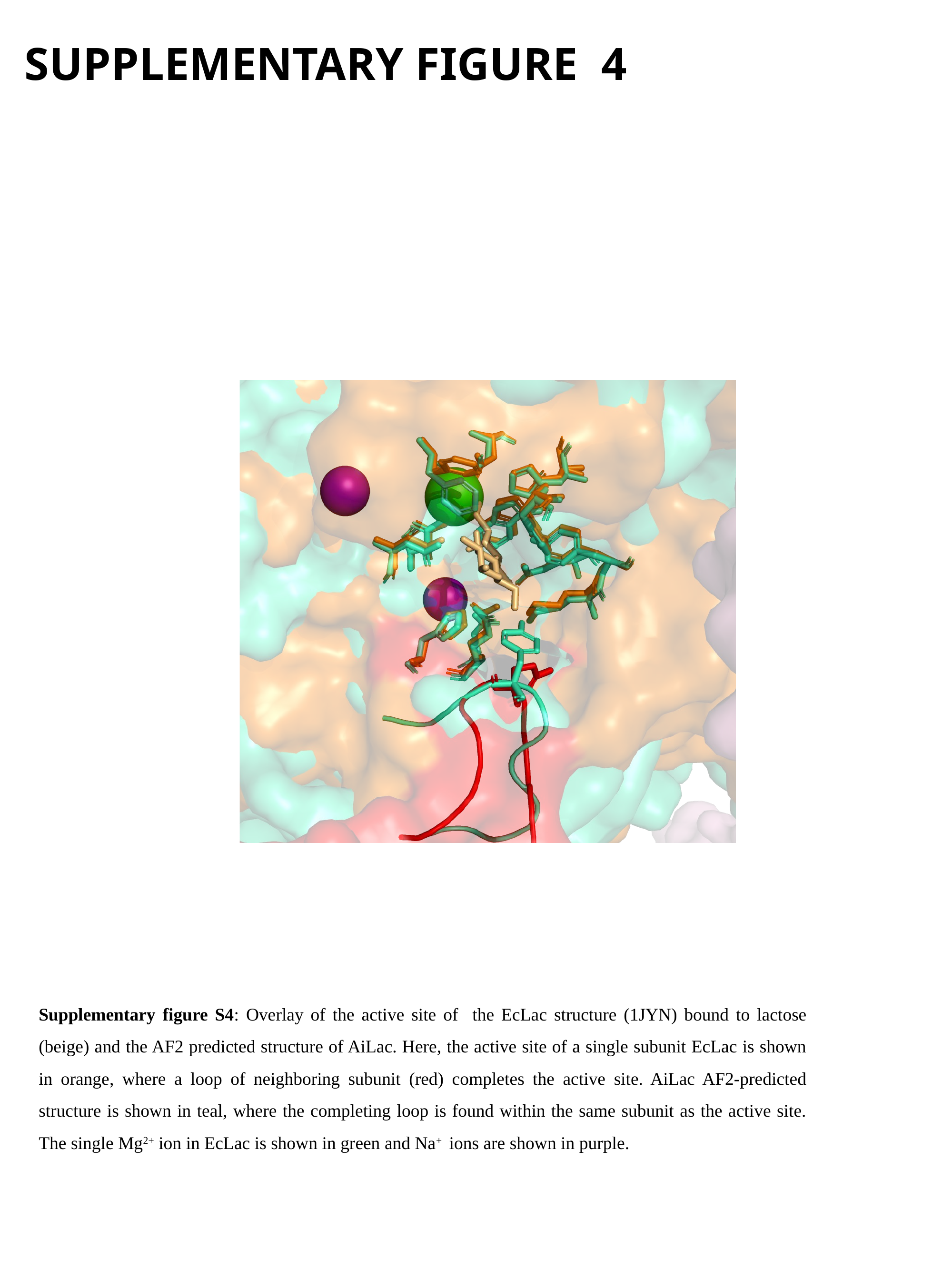

# Supplementary FIGURE 4
Supplementary figure S4: Overlay of the active site of the EcLac structure (1JYN) bound to lactose (beige) and the AF2 predicted structure of AiLac. Here, the active site of a single subunit EcLac is shown in orange, where a loop of neighboring subunit (red) completes the active site. AiLac AF2-predicted structure is shown in teal, where the completing loop is found within the same subunit as the active site. The single Mg2+ ion in EcLac is shown in green and Na+ ions are shown in purple.
07/04/202530/05/2022

## Slide 5
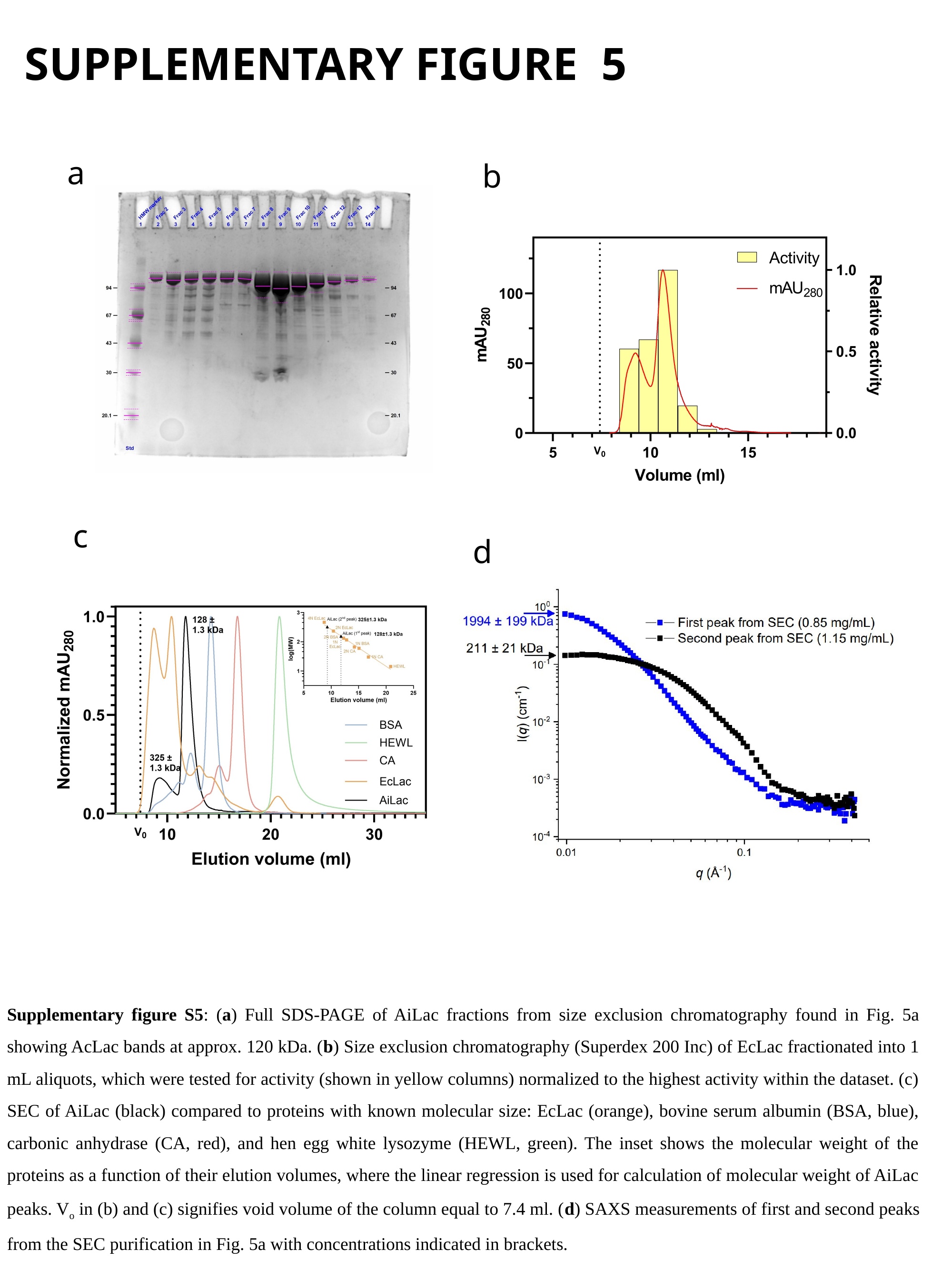

# Supplementary FIGURE 5
07/04/202530/05/2022
a
b
c
d
Supplementary figure S5: (a) Full SDS-PAGE of AiLac fractions from size exclusion chromatography found in Fig. 5a showing AcLac bands at approx. 120 kDa. (b) Size exclusion chromatography (Superdex 200 Inc) of EcLac fractionated into 1 mL aliquots, which were tested for activity (shown in yellow columns) normalized to the highest activity within the dataset. (c) SEC of AiLac (black) compared to proteins with known molecular size: EcLac (orange), bovine serum albumin (BSA, blue), carbonic anhydrase (CA, red), and hen egg white lysozyme (HEWL, green). The inset shows the molecular weight of the proteins as a function of their elution volumes, where the linear regression is used for calculation of molecular weight of AiLac peaks. Vo in (b) and (c) signifies void volume of the column equal to 7.4 ml. (d) SAXS measurements of first and second peaks from the SEC purification in Fig. 5a with concentrations indicated in brackets.

## Slide 6
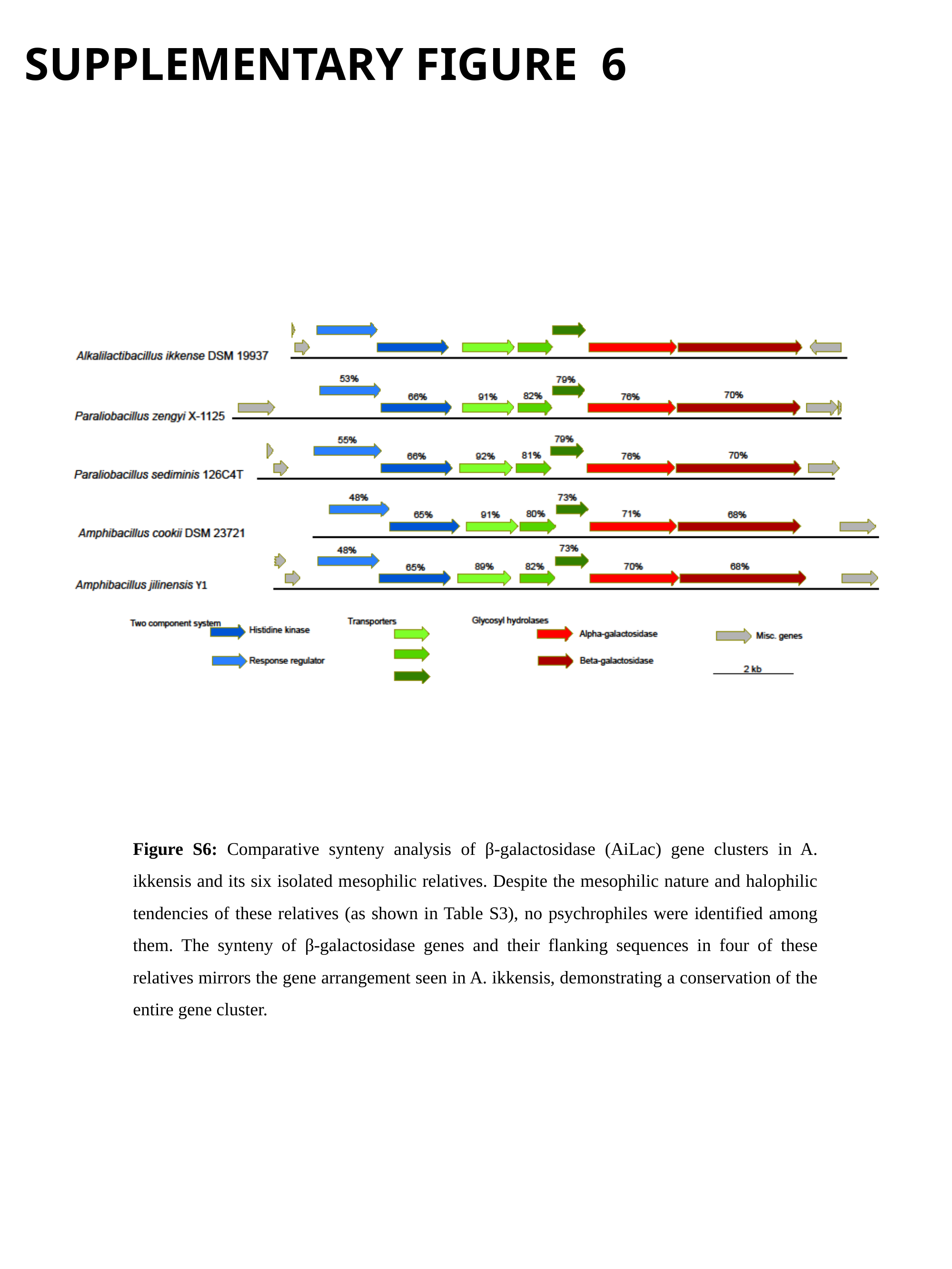

# Supplementary FIGURE 6
Figure S6: Comparative synteny analysis of β-galactosidase (AiLac) gene clusters in A. ikkensis and its six isolated mesophilic relatives. Despite the mesophilic nature and halophilic tendencies of these relatives (as shown in Table S3), no psychrophiles were identified among them. The synteny of β-galactosidase genes and their flanking sequences in four of these relatives mirrors the gene arrangement seen in A. ikkensis, demonstrating a conservation of the entire gene cluster.
07/04/202530/05/2022

## Slide 7
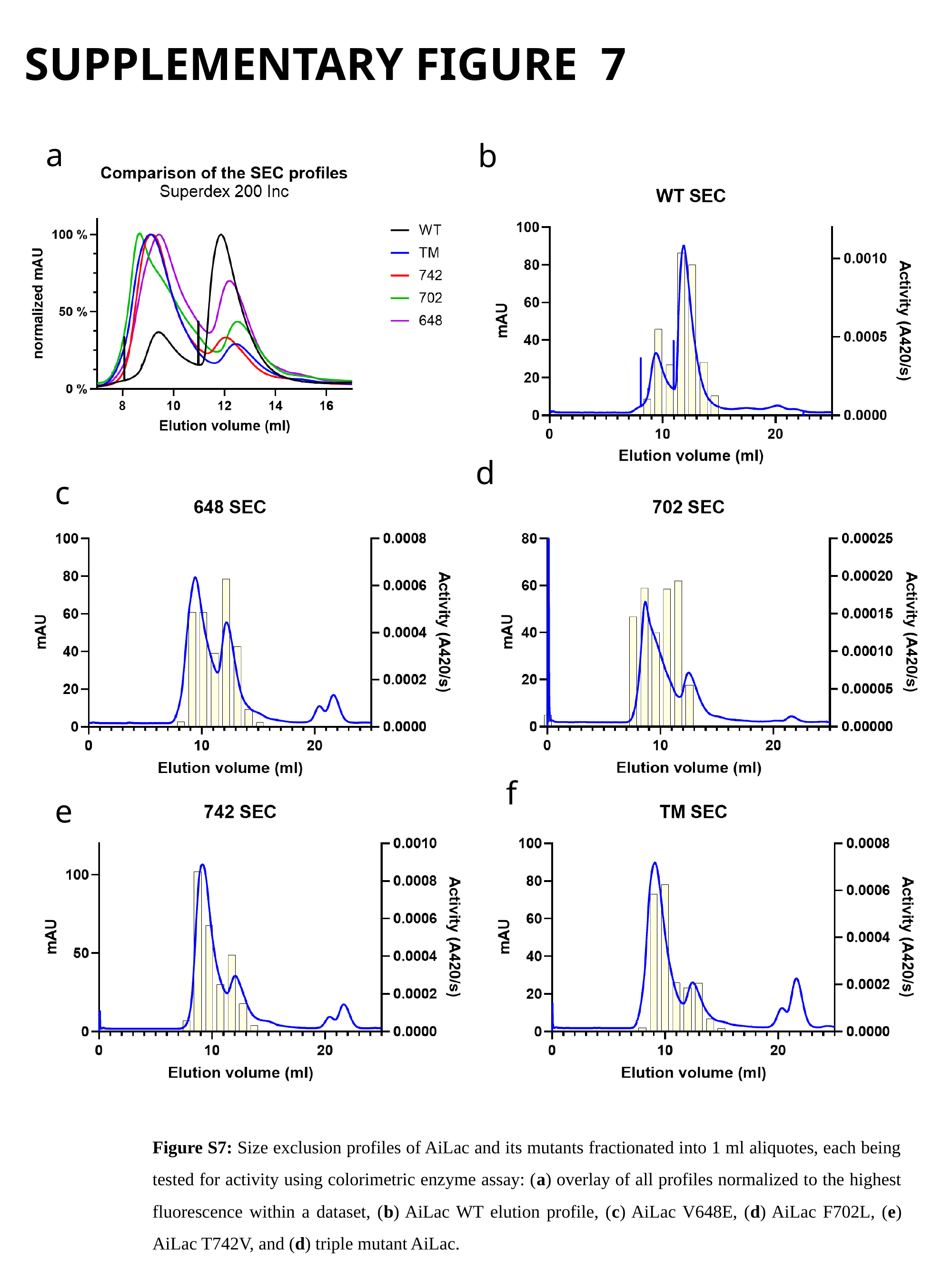

# Supplementary FIGURE 7
a
b
d
c
f
e
Figure S7: Size exclusion profiles of AiLac and its mutants fractionated into 1 ml aliquotes, each being tested for activity using colorimetric enzyme assay: (a) overlay of all profiles normalized to the highest fluorescence within a dataset, (b) AiLac WT elution profile, (c) AiLac V648E, (d) AiLac F702L, (e) AiLac T742V, and (d) triple mutant AiLac.
07/04/202530/05/2022

## Slide 8
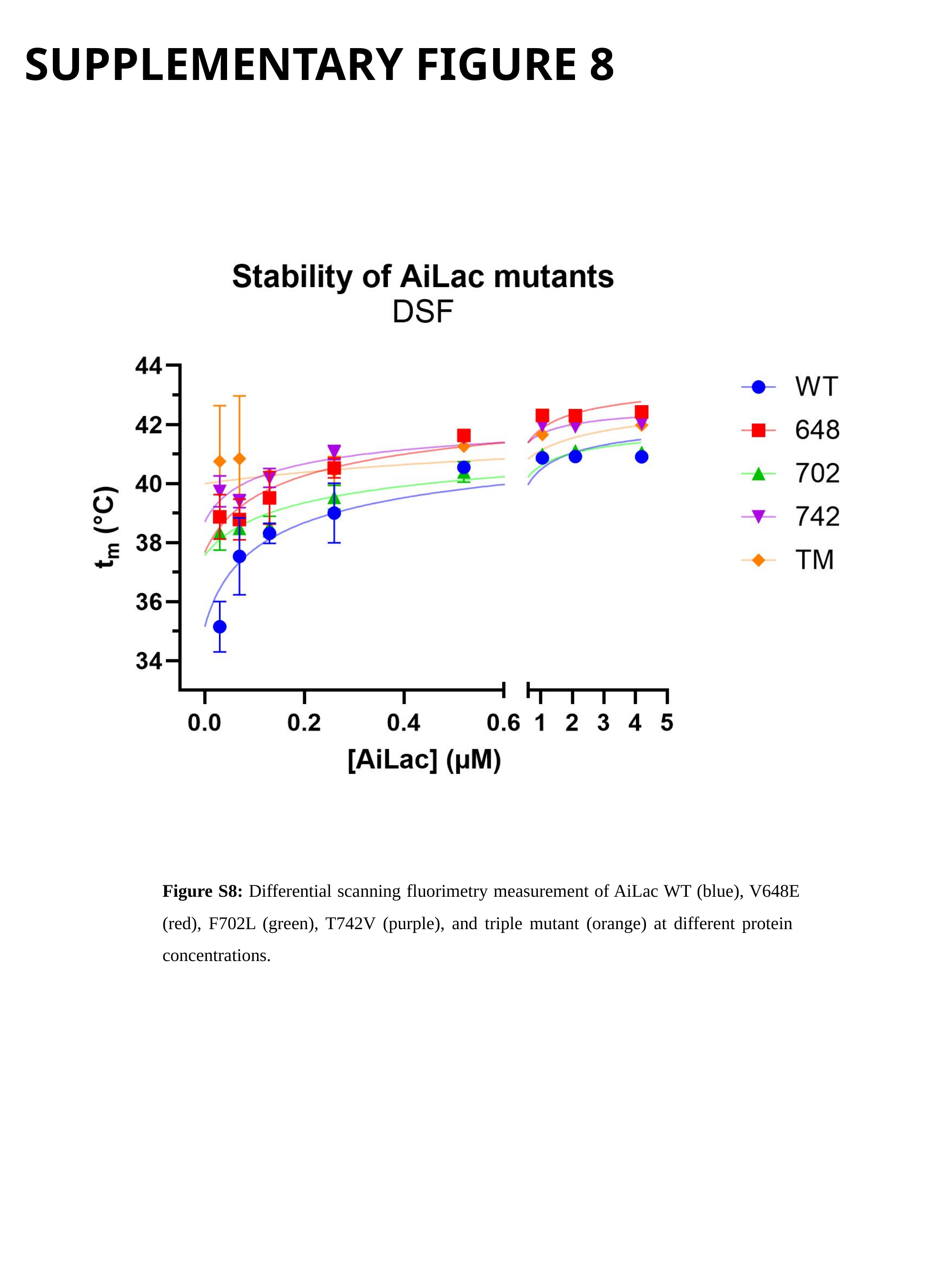

# Supplementary FIGURE 8
Figure S8: Differential scanning fluorimetry measurement of AiLac WT (blue), V648E (red), F702L (green), T742V (purple), and triple mutant (orange) at different protein concentrations.
07/04/202530/05/2022
