# Supplementary material for: Quaternary stabilization of a GH2 β‐galactosidase from the psychrophile A. ikkensis, a flexible and unstable dimeric enzyme
Source: Protein Sci. 2025 Apr 25;34(5):e70141. doi: 10.1002/pro.70141 (PMC12023411; doi:10.1002/pro.70141)
Supplement: Supplementary file 2 — Table S1: R g values obtained from SAXS data for AiLac at different temperatures (left columns, SAXS data in Figure S2b) and [Urea] (right column, SAXS data in Figure S2c)a. Table S2: Fit values for AF2 dimer to measured SAXS data along with the individual and average results from the Monte Carlo simulations. ρhydration describes the contribution from the hydration layer and can have a value between 0.2 and 1.3 depending on the protein. Table S3: Overview of the six isolated A. ikkensis relatives that also produce β‐galactosidases. [file PRO-34-e70141-s001.docx]

**Table S1:** *R*g values obtained from SAXS data for AiLac at different temperatures (left columns, SAXS data in Fig. S2b) and [Urea] (right column, SAXS data in Fig. S2c)^a^.

| *t* (°C) | Rg (nm) |  | [Urea] (M) | *R*g (nm) |
| --- | --- | --- | --- | --- |
| 10 | 4.4 |  | 0 | 5.2 |
| 15 | 4.4 |  | 0.25 | 11.9 |
| 20 | 4.4 |  | 0.5 | 12.3 |
| 25 | 4.3 |  | 0.75 | 13.7 |
| 30 | 7.4 |  | 1 | 13.7 |
| 35 | 12.0 |  | 1.25 | 13.7 |
| 40 | 12.2 |  | 1.5 | 13.9 |
| 45 | 12.8 |  |  |  |

Notes:

^a^ All experiments performed in in PBS pH 7.0 with 0.1 mM Mg^2+^.

**Table S2:** Fit values for AF2 dimer to measured SAXS data along with the individual and average results from the Monte Carlo simulations. ρhydration describes the contribution from the hydration layer and can have a value between 0.2 and 1.3 depending on the protein.

|  | χ² | *c* (mg/mL) | ρhydration |
| --- | --- | --- | --- |
| AF2 dimer | 13.8 | 1.046 ± 0.009 | 0.48 ± 0.01 |
| Simulations | | | |
|  | χ² | *c* (mg/mL) | RMSD (Å) |
| 1 | 2.92 | 1.013 | 3.1 |
| 2 | 3.00 | 1.012 | 2.8 |
| 3 | 2.92 | 1.010 | 2.8 |
| 4 | 2.81 | 1.013 | 3.5 |
| 5 | 2.87 | 1.013 | 2.8 |
| 6 | 2.66 | 1.016 | 4.2 |
| 7 | 2.98 | 1.009 | 2.9 |
| 8 | 2.98 | 1.011 | 2.5 |
| 9 | 2.91 | 1.011 | 2.5 |
| 10 | 2.80 | 1.013 | 3.2 |
| Average | 2.89 ± 0.10 | 1.012 ± 0.002 | 3.0 ± 0.5 |

**Table S3:** Overview of the six isolated *A. ikkensis* relatives that also produce β-galactosidases.

| **Species** | **% Identity to AiLac** | **Isolation site** | **Growth conditions** | **Reference** |
| --- | --- | --- | --- | --- |
| *Paraliobacillus quinghaiensis* YIM-C158T | 75 | Sediment of a salt lake in the Qaidam Basin, north-west China | Strain YIM-C158T grew in the presence of 1-20 % (w/v) NaCl and pH 6.0-10.0, with optimum growth at 5 % (w/v) NaCl and pH 8.0. The strain grew at 4-50 degrees C, with  optimum growth at 37 degrees C. | (Chen, Cui et al. 2009) |
| *Paraliobacillus* *zengyii* X- 1125T | 70 | Faeces of Tibetan antelopes | 28 °C, 3 % (w/v) NaCl and pH 7.5, | (Wang, Yang et al. 2019) |
| *Paraliobacillus sediminis* | 70 | Sediment from the East China Sea | Optimal growth occurred at 28-30 °C, pH 7.0-7.5 and in  the presence of 3-5 % (w/v) NaCl | (Cao, Guo et al. 2017) |
| *Amphibacillus cookii* | 68 | Southern Arm of Great Salt Lake, Utah | between 14.5 and 47 °C (optimum 39 °C), in the pH(37  °C) range 6.5-10.3 (optimum pH(37 °C) 8.0), and between  0.1 and 4.5 M Na(+) (optimum  0.9 M Na(+)). | (Pugin, Blamey et al. 2012) |
| *Amphibacillus xylanus* NBRC 15112 | 68 | Lake sediment in Japan | Opt 37 C (25-44 C), pH 8.0-  10.0 (no growth 7.0); 3.0% NaCl, no growth 6.0 | (An, Ishikawa et al. 2007) |
| *Amphibacillus jilinensis* Y1 | 68 | Sediment from a soda lake, China  Jilin Province | The optimum pH for growth was 9.0, with a range of pH 7.5-10.5. No growth occurred at pH 7.0 or 11.0. The strain was mesophilic, with a  temperature range of 15-45 °C and optimum growth at 32 °C. | (Wu, Zheng et al. 2010) |

**References:**

An, S. Y., S. Ishikawa, H. Kasai, K. Goto and A. Yokota (2007). "Amphibacillus sediminis sp. nov., an endospore-forming bacterium isolated from lake sediment in Japan." Int J Syst Evol Microbiol **57**(Pt 11): 2489-2492.

Cao, W. R., L. Y. Guo, Z. J. Du, A. Das, G. Saren, M. Y. Jiang, C. A. Dunlap, A. P. Rooney, X. K. Yu and T. G. Li (2017). "Paraliobacillus sediminis sp. nov., isolated from East China sea sediment." Int J Syst Evol Microbiol **67** (5): 1577-1581.

Chen, Y. G., X. L. Cui, Y. Q. Zhang, W. J. Li, Y. X. Wang, L. H. Xu, M. L. Wen, Q. Peng and C. L. Jiang (2009). "Paraliobacillus quinghaiensis sp. nov., isolated from salt-lake sediment in China." Int J Syst Evol Microbiol **59** (Pt 1): 28-33.

Pugin, B., J. M. Blamey, B. K. Baxter and J. Wiegel (2012). "Amphibacillus cookii sp. nov., a facultatively aerobic, spore-forming, moderately halophilic, alkalithermotolerant bacterium." Int J Syst Evol Microbiol **62** (Pt 9): 2090-2096.

Wang, X., J. Yang, S. Lu, X. H. Lai, D. Jin, J. Pu, L. Niu, W. Zhu, J. Liang, Y. Huang, B. Wang, X. Wu, H. Liang and J. Xu (2019). "Paraliobacillus zengyii sp. nov., a slightly halophilic and extremely halotolerant bacterium isolated from Tibetan antelope faeces." Int J Syst Evol Microbiol **69** (5): 1426-1432.

Wu, X. Y., G. Zheng, W. W. Zhang, X. W. Xu, M. Wu and X. F. Zhu (2010). "Amphibacillus jilinensis sp. nov., a facultatively anaerobic, alkaliphilic bacillus from a soda lake." Int J Syst Evol Microbiol **60** (Pt 11): 2540-2543.
